# Genes Linking Copper Trafficking and Homeostasis to the Biogenesis and Activity of the *cbb*_3_-Type Cytochrome *c* Oxidase in the Enteric Pathogen *Campylobacter jejuni*

**DOI:** 10.3389/fmicb.2021.683260

**Published:** 2021-06-25

**Authors:** Nitanshu Garg, Aidan J. Taylor, Federica Pastorelli, Sarah E. Flannery, Phillip J. Jackson, Matthew P. Johnson, David J. Kelly

**Affiliations:** Department of Molecular Biology and Biotechnology, The University of Sheffield, Sheffield, United Kingdom

**Keywords:** copper, homeostasis, periplasm, chaperone, supercomplex

## Abstract

Bacterial C-type haem-copper oxidases in the *cbb*_3_ family are widespread in microaerophiles, which exploit their high oxygen-binding affinity for growth in microoxic niches. In microaerophilic pathogens, C-type oxidases can be essential for infection, yet little is known about their biogenesis compared to model bacteria. Here, we have identified genes involved in *cbb*_3_-oxidase (Cco) assembly and activity in the Gram-negative pathogen *Campylobacter jejuni*, the commonest cause of human food-borne bacterial gastroenteritis. Several genes of unknown function downstream of the oxidase structural genes *ccoNOQP* were shown to be essential (*cj1483c* and *cj1486c*) or important (*cj1484c* and *cj1485c*) for Cco activity; Cj1483 is a CcoH homologue, but Cj1484 (designated CcoZ) has structural similarity to MSMEG_4692, involved in Qcr-oxidase supercomplex formation in *Mycobacterium smegmatis*. Blue-native polyacrylamide gel electrophoresis of detergent solubilised membranes revealed three major bands, one of which contained CcoZ along with Qcr and oxidase subunits. Deletion of putative copper trafficking genes *ccoI* (*cj1155c*) and *ccoS* (*cj1154c*) abolished Cco activity, which was partially restored by addition of copper during growth, while inactivation of *cj0369c* encoding a CcoG homologue led to a partial reduction in Cco activity. Deletion of an operon encoding PCu_*A*_C (Cj0909) and Sco (Cj0911) periplasmic copper chaperone homologues reduced Cco activity, which was partially restored in the *cj0911* mutant by exogenous copper. Phenotypic analyses of gene deletions in the *cj1161c–1166c* cluster, encoding several genes involved in intracellular metal homeostasis, showed that inactivation of *copA* (*cj1161c*), or *copZ* (*cj1162c*) led to both elevated intracellular Cu and reduced Cco activity, effects exacerbated at high external Cu. Our work has therefore identified (i) additional Cco subunits, (ii) a previously uncharacterized set of genes linking copper trafficking and Cco activity, and (iii) connections with Cu homeostasis in this important pathogen.

## Introduction

Haem-copper oxidases (HCOs) are proton-translocating respiratory complexes widely distributed in both prokaryotes and eukaryotes that contain a bi-nuclear haem-copper site at which oxygen reduction occurs. Three families of HCO can be distinguished based primarily on the sequence similarity of the core subunits. Type A include the cytochrome *aa*_3_-type oxidases found in a range of aerobic bacteria and mitochondria, type B includes the *ba*_3_ oxidases only found in bacteria, and type C contains the *cbb*_3_-type oxidases, which have an integral c-type cytochrome subunit (Pereira et al., 2001). This type of enzyme has a high oxygen affinity and is typically found in a range of microaerophilic bacteria (Pitcher and Watmough, 2004).

Mechanistically, all HCOs operate using a redox loop mechanism for proton translocation across the membrane as well as pumping protons directly. However, while the A-type oxidases have two proton pathways (the D- and K-channels), which are used for water formation from oxygen and transmembrane proton pumping, respectively, the B- and C-types have a single proton pathway located in the same region as the K-channel of A-type oxidases (Buschmann et al., 2010). The stoichiometry of proton pumping by C-type oxidases has been controversial, and the best estimates indicate a H^+^/e ratio of only 1, which is lower than other types of HCO’s (Rauhamäki et al., 2012). However, working in combination with the quinol-cytochrome c reductase (Qcr) complex, the redox loop mechanism means that a higher overall H^+^/e ratio can be achieved during electron transfer from quinol to oxygen.

The redox cofactors in the *cbb*_3_ type of oxidase are bound to three subunits. CcoN contains a binuclear Cu_*B*_–haem *b*_3_ (high-spin) centre at which oxygen reduction occurs and an additional low-spin *b* haem. CcoO is a monohaem *c*-type cytochrome and CcoP is a dihaem *c*-type cytochrome. Electron transfer proceeds from the C-haems in CcoP to CcoO and then to oxygen via the redox centres in the CcoN subunit. Additional subunits have also been identified, with less clearly defined roles. CcoQ is a small protein needed for stabilising interactions of CcoP with the CcoNO core complex in some species (Peters et al., 2008) and which is commonly encoded in the same operon as the redox active subunits. Additional small subunits in addition to or in place of CcoQ are also found in complexes from different bacteria. The CcoH subunit is now known to be an integral protein in many, but not all purified complexes. Pawlik et al. (2010) demonstrated that biogenesis of CcoNOQP in *Rhodobacter capsulatus* proceeds via CcoQP and CcoNO sub-complexes, but in the absence of CcoH, neither the fully assembled CcoNOQP nor the CcoQP or CcoNO sub-complex were detectable. CcoH binds to CcoP via its transmembrane domain and stays tightly associated with the active, fully assembled complex. Type C oxidases from some *Pseudomonas* sp. can contain CcoM, a single transmembrane subunit required for complex stability but not activity (Kohlstaedt et al., 2016; Carvalheda and Pisliakov, 2017). CcoM and CcoQ share low sequence identity and it is thought they have different functions (Kohlstaedt et al., 2016; Carvalheda and Pisliakov, 2017).

A number of additional proteins have been found to be crucial for oxidase biogenesis and in particular for the export of copper to the periplasm and its insertion into the CcoN active site subunit. The *ccoG*, *ccoI*, and *ccoS* genes encode proteins involved in copper handling and export and their roles were first identified in *Bradyrhizobium japonicum* (Preisig et al., 1996) and further clarified in *R. capsulatus* (Koch et al., 2000). CcoG contains two cysteine-rich motifs that resemble those encountered in [4Fe–4S] cluster containing ferrodoxins, suggesting it has an oxidoreductase role (Preisig et al., 1996) and it has recently been shown to act as a cupric reductase (Marckmann et al., 2019). CcoI is a Cu-translocating P-type ATPase that transfers copper to the periplasm (Preisig et al., 1996; Koch et al., 2000). CcoS contains no characteristic motifs or homology to characterised proteins (Koch et al., 2000). Studies of the assembly of the *cbb*_3_-type cytochrome *c* oxidase in *R. capsulatus* showed that in a *ccoI* deletion strain, the sub-complex CcoNOQ was absent, although monomeric CcoP was still detectable. In the absence of CcoS, the complex was assembled but with no enzymatic activity (Kulajta et al., 2006).

In eukaryotic cells, two key proteins Sco1 and Cox17 are required for the correct assembly of cytochrome *c* oxidase (Dickinson et al., 2000; Mattatall et al., 2000). The Cox17 protein is believed to be the specific copper donor to the mitochondrial inner membrane bound Sco1 (Horng et al., 2004). Mattatall et al. (2000) reported the discovery of YpmQ, a bacterial homolog of Sco1. Deletion of *ypmQ* in *Bacillus subtilis* reduced the expression of cytochrome *c* oxidase, the level of which was recovered on substituting the growth medium with additional copper. In *Deinococcus radiodurans* and *Caulobacter crescentus*, Banci et al. (2005) found a class of proteins of unknown function (referred to as PCu_*A*_C) displaying a conserved gene neighbourhood to bacterial *sco1* genes, all sharing a potential metal binding motif H(M)X_10_MX_21_HXM, and potentially taking the role of Cox17 in bacteria, involving copper delivery to the Cu(A) center in the oxidase. Bacterial PCu_*A*_C and Sco protein encoding genes are widespread and often found in the same operon (Abriata et al., 2008). For example in *R. capsulatus*, SenC, a Sco1-homologue and PccA, a PCu_*A*_C-like periplasmic chaperone, have been identified. PccA has been demonstrated as a Cu-binding protein with a preference for Cu(I), which is required for efficient *cbb*_3_-Cox assembly, in particular, at low Cu concentrations (Trasnea et al., 2016). Cu is transferred from PccA to SenC and vice versa at similar levels, constituting a Cu relay system that facilitates Cu delivery to *cbb*_3_-type Cco (Trasnea et al., 2018). In the absence of PccA, SenC still obtains Cu that is exported by CcoI and facilitates Cco assembly, although with lower efficiency (Trasnea et al., 2018).

In contrast to the extensive information available for oxidase biogenesis and function in well studied model bacteria such as *R. capsulatus*, much less is known about the oxidases of divergent unrelated bacteria, such as those of the Campylobacterota (formerly Epsilonproteobacteria), containing well known human pathogens in the genera *Campylobacter*, *Helicobacter*, and *Arcobacter*. The microaerophilic *C. jejuni* is a normal commensal of birds, including poultry, where undercooked chicken is the main source of human infection. It is the most frequent cause of food-borne bacterial gastroenteritis worldwide, leading to an enormous disease buden and serious sequelae such as Guillain-Barré syndrome (O’Brien, 2017). Increasing antibiotic resistance is also a major problem and *C. jejuni* has been designated a WHO priority pathogen for study.

**FIGURE 1:**
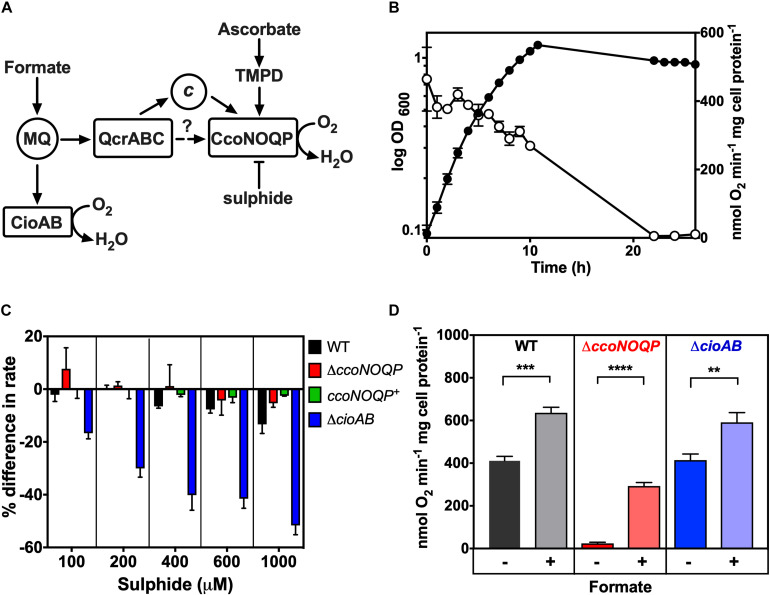
Characterisation of cytochrome *c* oxidase activity in *Campylobacter jejuni*. **(A)** Simplified schematic of electron transport pathways to oxygen. Formate is a major physiological electron donor to the menaquinone (MQ) pool, where electrons can be directed either to the quinol oxidase CioAB or to the QcrABC complex and on to the *cbb*_3_-type cytochrome *c* oxidase CcoNOQP. Ascorbate reduced TMPD specifically acts as a non-physiological electron donor to the CcoNOQP oxidase. Sulphide inhibits activity (—|) **(B)** Cco activity in wild-type cells grown in Mueller-Hinton medium at different stages of growth, measured as oxygen uptake in a Clark type electrode with ascorbate plus TMPD as electron donor. The data points show the mean rate of three independent experiments with the errors bars showing SD. In many cases the error bars are too small to be seen. Closed circles, OD measured at 600 nm; open circles, specific rate of ascorbate/TMPD driven oxygen uptake. **(C)** Effect of increasing sulphide concentrations on the rate of ascorbate/TMPD oxidase activity in wild-type (black bars), *ccoNOQP* deletion mutant (red bars), *ccoNOQP* complemented strain (*ccoNOQP*^+^; green bars) and *cioAB* mutant (blue bars). The results are shown as the mean% difference in the rate compared to the rate without any sulphide addition. Error bars show SD from three independent experiments. **(D)** Cco activity measured with ascorbate/TMPD without (**−**) and with (**+)** 20 mM sodium formate in the assay. Wild type (black bars), Δ*ccoNOQP* (red bars) and Δ*cioAB* (blue bars) cells were grown to mid-exponential phase in Mueller-Hinton medium. The data are the average of triplicate cultures with error bars showing SD. Student’s *t*-tests were performed to test significance (0.01 > **p > 0.001; 0.001 > ***p > 0.0001; 0.0001 > ****p > 0.00001). Raw data for Figures 1–7 can be found in Supplementary Tables 1, 2.

A *cbb*_3_-type cytochrome c oxidase and a cytochrome bd-type quinol oxidase (Figure 1A) are present in the *C. jejuni* reference strain NCTC 11168 (Parkhill et al., 2000; Taylor and Kelly, 2019) and both oxidases are highly conserved in all other sequenced strains. The cytochrome c oxidase in *C. jejuni* is currently thought to be encoded by *ccoNOQP* genes (*cj1490c*–*cj1487c*; Parkhill et al., 2000) and has been shown to have a very high affinity for oxygen (Jackson et al., 2007). A *ccoN* mutant was constructed by Weingarten et al. (2008) and reported to be highly sensitive to oxygen and to exhibit a microaerobic growth defect. Importantly, this mutant was unable to colonise chicks (Weingarten et al., 2008) indicating a crucial role for the Cco enzyme in energy conservation *in vivo*. A *ccoNOQP* operon deletion mutant constructed by Liu and Kelly (2015) also had an aerobic growth defect but did not seem to be more sensitive to oxygen than the wild-type and measurements using a fluorescent probe did not show accumulation of reactive oxygen species (ROS). A study of gene expression in *C. jejuni* growing in the chick caecum also indicated the importance of this oxidase in host colonisation: the *cco* gene cluster was up-regulated about fourfold, suggesting the bacteria are present in a microaerobic niche (Woodall et al., 2005).

Only the *ccoNOQP* structural genes for the *cbb*_3_-oxidase have thus far been identified in *C. jejuni* (Parkhill et al., 2000; Taylor and Kelly, 2019) and in particular it is unknown which additional genes/proteins are required for insertion of copper to form an active enzyme. In this work we have tested biogenesis gene function predictions based on homology and gene context by phenotypic analyses of deletion mutants in strain NCTC 11168. Table 1 provides a list of the genes studied. In addition to characterizing the function of CcoG, H, I, S, Sco, and PcuC homologues by such mutant analyses, we also show that several novel genes are required for oxidase activity. The product of one of these (designated CcoZ) has structural similarity to actinobacterial proteins involved in oxidase-cytochrome *bc* supercomplex formation. Preliminary evidence such an association is presented.

**TABLE 1:** Genes tested in this work for their role in oxidase assembly, activity and copper homeostasis.

*Campylobacter jejuni* locus tag	Gene name	Gene product function	Predicted cellular location
*cj0369c*	*ccoG*	Cupric reductase for *cbb*_3_ oxidase biogenesis	Inner membrane anchored; cytoplasm facing
*cj0908*	*–*	Unknown	Inner membrane anchored; periplasm facing
*cj0909*	*pcuC*	Possible periplasmic Cu chaperone. Contains DUF461	Periplasm
*cj0910*	*–*	Unknown	Inner membrane anchored; periplasm facing
*cj0911*	*sco1*	Periplasmic Cu chaperone	Inner membrane anchored; Periplasm facing
*cj1154c*	*ccoS*	*cbb*_3_-oxidase biogenesis protein	Inner membrane anchored
*cj1155c*	*ccoI*	*cbb*_3_-oxidase biogenesis protein; Cu exporting ATPase	Inner membrane
*cj1161c*	*copA*	Cu exporting ATPase	Inner membrane
*cj1162c*	*copZ*	Cu chaperone	Cytoplasm
*cj1163c*	*czcD*	Zn exporting ATPase	Inner membrane
*cj1164c*	*–*	Unknown; Zn finger protein	Cytoplasm
*cj1165c*	*–*	Unknown; DUF1212, possible solute transporter	Inner membrane
*cj1166c*	*–*	Unknown; DUF1212, possible solute transporter	Inner membrane
*cj1482c*	*–*	Unknown; DUF386. Possible DNA helicase	Cytoplasm
*cj1483c*	*ccoH*	Oxidase subunit	Inner membrane
*cj1484c*	*ccoZ*	Unknown; DUF5130	Inner membrane
*cj1485c*	*ccoY*	Unknown	Inner membrane
*cj1486c*	*ccoX*	Unknown; DUF4006	Inner membrane
*cj1487c*	*ccoP*	Oxidase subunit; cytochrome c	Inner membrane; periplasm facing
*cj1488c*	*ccoQ*	Oxidase subunit	Inner membrane
*cj1489c*	*ccoO*	Oxidase subunit; cytochrome c	Inner membrane; periplasm facing
*cj1490c*	*ccoN*	Oxidase subunit; Cu-heam protein	Inner membrane

*DUF, domain of unknown function. Cellular location of gene products was inferred using sequence analysis and topology prediction by Signal P and TMHMM servers.*

## Materials and Methods

### Strains and Culture Conditions

*Campylobacter jejuni* strains were routinely grown at 42°C under microaerobic conditions [10% (v/v) O_2_, 5% (v/v) CO_2_, and 85% (v/v) N_2_] in a MACS growth cabinet (Don Whitley Scientific, Shipley, United Kingdom). Bacterial cells, from frozen stocks, were streaked on Columbia blood agar plates (with appropriate antibiotics) and incubated overnight in the MACS growth cabinet. Material from plates were used for inoculating 25–50 ml starter cultures in Mueller-Hinton broth supplemented with 20 mM L-serine and 10 μg ml^–1^ each of vancomycin and amphotericin B (MHS broth). Cultures were incubated on a shaker at 150 rpm in the MACS growth cabinet for 6–12 h followed by inoculation into growth cultures to an initial OD_600_ of ∼0.1. For mutant selection, kanamycin was used at 50 μg ml^–1^. Minimum Essential Medium alpha (MeMα), (nucleosides added, no phenol red; Life Science technologies) was also used to grow *C. jejuni* for some experiments (see text). MeMα was supplemented with 40 μM FeSO_4_, 20 mM Sodium Pyruvate and 20 mM L-Serine.

### Copper Sensitivity Assay

*Campylobacter jejuni* starter cultures were grown as above. Cell pellets were washed by resuspending in 25 ml MeMα minimal media and centrifuged at 12,000×*g* for 4 min at 42°C. Cells were resuspended in MeMα minimal media and added to 5 ml MeMα (equilibrated for 4 h in a microaerobic environment at 42°C with shaking) in six well plates to make the starting OD_600_ ∼0.1. The media was supplemented with 0.1–5.0 mM copper sulphate (CuSO_4_) (Sigma-Aldrich). The six well plates were incubated in a microaerobic environment at 42°C with shaking at 160 rpm until cells reached stationary phase at which point the OD_600_ was measured.

### Cytochrome *c* Oxidase Activity

*cbb*_3_-oxidase activity was measured as the rate of oxygen consumption of cell suspensions in a Clark-type oxygen electrode using 0.25 mM tetramethyl-*p*-phenylenediamine (TMPD) plus 1 mM sodium ascorbate as electron donor, calibrated using air-saturated 25 mM sodium phosphate buffer (pH 7.4) (220 nmol dissolved O_2_ ml^–1^ at 42°C). For wild-type and mutant comparisons, 25 ml *C. jejuni* cultures were grown until exponential phase in MHS without additions or with 0.25 mM copper sulphate, as required. Cells were pelleted and re-suspended in ice cold 1 ml 20 mM sodium phosphate buffer (pH 7.4) and kept on ice. Total protein concentration of the cell suspension was determined by Lowry assay and the specific rate of oxidation was calculated as nmol oxygen consumed min^–1^ mg^–1^ total protein.

### Inductively Coupled Plasma Mass Spectrometry of Cell Samples

Fractionation of *C. jejuni* cell compartments with minimal cross-contamination is challenging so we determined total intracellular metal contents using whole cell samples. Biological triplicate cultures of *C. jejuni* cells were grown in 500 ml MHS broth and cells were harvested at stationary phase by centrifuging at 17,700×*g* for 20 min at 4°C. Cell pellets were re-suspended in 10 ml wash buffer (10 mM HEPES, 0.5 M Sorbitol and 100 μM EDTA, pH 7.5) and centrifuged as above. The wash steps were repeated three times but in the third wash, EDTA was omitted from the wash buffer. After the final centrifugation the supernatants were discarded and cell pellets was resuspended in 1 ml concentrated HNO_3_ (65% v/v) by vortexing. Samples were left in acid overnight and then analysed at the University of Sheffield Inductively Coupled Plasma Mass Spectrometry (ICP-MS) facility.

### Isothermal Assembly Cloning and Allelic Exchange Mutagenesis

Isothermal assembly (ISA) cloning was used to generate mutant plasmids for transformation into *C. jejuni*, in order to delete specific genes by allelic exchange mutagenesis, replacing most of the coding region with a kanamycin resistance cassette derived from pJMK30 (van Vliet et al., 1998). The ISA reactions were performed by assembling 4 PCR amplified fragments; *Hin*cII digested pGEM3Zf(–) vector, two regions of ∼500 bp flanking the gene to be deleted, such that only the first few and last few codons of the gene were retained, and the kanamycin resistance cassette to be inserted between the two flanking regions. The kanamycin resistance cassette was amplified from pJMK30 using primers Kan F and Kan R (Table 2). The left flanking region of the gene to be deleted was amplified using primers F1 F and F1 KR (Table 2) and the right flanking region was amplified by F2 KF and F2 R. The F1 F and F2 R primers contain 30 bp adapters for the pGEM3zf(–) vector cut with *Hin*cII and the F1 R and F2 F primers contain 30 bp adapters for the kanamycin resistance cassette. All PCR amplifications were performed with Phusion polymerase (NEB). An ISA mastermix was prepared by mixing 40 μl 5 X ISA buffer, 0.125 μl T5 exonuclease (Cambio, United Kingdom), 2.5 μl Phusion polymerase (NEB) and 20 μl Taq ligase (NEB) and made to a total volume of 150 μl with dH_2_O. 5 X ISA buffer consists of 25% (w/v) polyethylene glycol [PEG-8000], 500 mM *Tris*–HCl pH 7.5, 50 mM MgCl_2_, 50 mM dithiothreitol [DTT], 1 mM of each dNTP, and 5 mM NAD. pGEM3Zf (–) was digested with *Hin*cII and phosphatase treated. The PCR fragments for the ISA reaction were combined in equimolar concentration with amounts ranging from 10 to 100 ng each, with the final volume of the mixture not exceeding 5 μl. A 15 μl ISA mastermix aliquot was thawed on ice and the mixture was added to it with the final volume made up to 20 μl with dH_2_O. The reaction was incubated at 50°C for 1 h. The resulting DNA was immediately used to transform competent *Escherichia coli* DH5α cells, which were then grown on LB+ kanamycin agar plates. Colonies were screened by PCR using M13 primers and correct assembly of plasmids was confirmed by automated DNA sequencing (Core Genomic Facility, University of Sheffield Medical School, United Kingdom). Plasmids were electroporated into *C. jejuni*, cells plated out on non-selective Columbia agar plates and incubated overnight in microaerobic conditions at 42°C. The growth was harvested and transferred to selective Columbia agar plates (with kanamycin) and incubated as above for 2–4 days. Colonies were checked by colony PCR with a combination of different screening and antibiotic cassette primers according to Table 2.

**TABLE 2:** Primers used in this work.

*Campylobacter jejuni* NCTC 11168 gene deletion ISA primers 5′–3′	
Cj0908 ISA F1 F	GAGCTCGGTACCCGGGGATCCTCTAGAGTCACC TACTGGAGTTTTCAAATCTCC
Cj0908 ISA F1 KR	AAGCTGTCAAACATGAGAACCAAGGAGAATTATA GACAACCTTTTAAAACAAGCC
Cj0911 ISA F1 F	GAGCTCGGTACCCGGGGATCCTCTAGAGTCTTA ATAATCACAAAACCATAGAGTTAAAA
Cj0911 ISA F1 KR	AAGCTGTCAAACATGAGAACCAAGGAGAATTTT TTTAGCTCAAAATCAAAATAAAA
Cj0911 ISA F2 KF	GAATTGTTTTAGTACCTAGCCAAGGTGTGCTTA TACTTAAGCAAAAAGATCGGTT
Cj0911 ISA F2 R	AGAATACTCAAGCTTGCATGCCTGCAGGTCGTT TCTCAATTTGAAAATATTTCAAATA
CJ1154C ISA F1 F	GAGCTCGGTACCCGGGGATCCTCTAGAGTCAAG AGAAGGTGCAAAAGAGC
CJ1154C ISA F1 R	AAGCTGTCAAACATGAGAACCAAGGAGAATTCA TGATTATACTATTCATTCTT
CJ1154C ISA F2 F	GAATTGTTTTAGTACCTAGCCAAGGTGTGCTTC TTAAAAAGAAAAGCCTT
CJ1154C ISA F2 R	AGAATACTCAAGCTTGCATGCCTGCAGGTCCAC CGTCTCTATCTAAAAATA
CJ1155C ISA F1 F	GAGCTCGGTACCCGGGGATCCTCTAGAGTCATC GCTTCGATTTTTAAAAG
CJ1155C ISA F1 R	AAGCTGTCAAACATGAGAACCAAGGAGAATTGC AATGTTCACATTTCATA
CJ1155C ISA F2 F	GAATTGTTTTAGTACCTAGCCAAGGTGTGCAGG ATTAAAGAATGAATAGTATAA
CJ1155C ISA F2 R	AGAATACTCAAGCTTGCATGCCTGCAGGTCCAC CGTCTCTATCTAAAAATA
CJ0369C ISA F1 F	GAGCTCGGTACCCGGGGATCCTCTAGAGTCACC TATCATACCAATTTGAATGATT
CJ0369C ISA F1 R	AAGCTGTCAAACATGAGAACCAAGGAGAATGTG TCCTTGCATTTTTAGACC
CJ0369C ISA F2 F	GAATTGTTTTAGTACCTAGCCAAGGTGTGCAAA AATGAACTCAAATAGAACACCATCAC
CJ0369C ISA F2 R	AGAATACTCAAGCTTGCATGCCTGCAGGTCAGG AGCGTTAATTAAAACATTAAGAT
CJ1483C ISA F1 F	GAGCTCGGTACCCGGGGATCCTCTAGAGTCTTT TAATGAAAATATTTTAAGTCAAAAAGTAA
CJ1483C ISA F1 R	AAGCTGTCAAACATGAGAACCAAGGAGAATAAT TATAGCCAAAAGTGAAAGCA
CJ1483C ISA F2 F	GAATTGTTTTAGTACCTAGCCAAGGTGTGCGAA ACAGTGGGTTTTTTTAGCT
CJ1483C ISA F2 R	AGAATACTCAAGCTTGCATGCCTGCAGGTCTAC TTAAAAAACCTTGCAAATCATAA
CJ1486C ISA F1 F	GAGCTCGGTACCCGGGGATCCTCTAGAGTCCAA TGGGTCAAAATATCTTTTTAGT
CJ1486C ISA F1 R	AAGCTGTCAAACATGAGAACCAAGGAGAATTCA CCCCTTGAAAGAGAAAT
CJ1486C ISA F2 F	GAATTGTTTTAGTACCTAGCCAAGGTGTGCGTA AGGCTTTGGAATATTTAATTGTT
CJ1486C ISA F2 R	AGAATACTCAAGCTTGCATGCCTGCAGGTCGCA GCATTATAAATATCACCTTTATTTG
CJ1485C ISA F1 F	GAGCTCGGTACCCGGGGATCCTCTAGAGTCATG TAGCAAAAGATCTTTCAGCT
CJ1485C ISA F1 R	AAGCTGTCAAACATGAGAACCAAGGAGAATTGT GCTTCCTTTATACTTCTATGATC
CJ1485C ISA F2 F	GAATTGTTTTAGTACCTAGCCAAGGTGTGCAAA AAATATTACAAGATGGCTTTTTT
CJ1485C ISA F2 R	AGAATACTCAAGCTTGCATGCCTGCAGGTCTAT CACGATTTGCATTACCTATAGA
CJ1484C ISA F1 F	GAGCTCGGTACCCGGGGATCCTCTAGAGTCCCA TGGAGAAGATGGAAAAG
CJ1484C ISA F1 R	AAGCTGTCAAACATGAGAACCAAGGAGAATCTA AAAAAAAGCCATCTTGTAATATTTT
CJ1484C ISA F2 F	GAATTGTTTTAGTACCTAGCCAAGGTGTGCACA AAGAAGAATAAAGAGGAAAAAAA
CJ1484C ISA F2 R	AGAATACTCAAGCTTGCATGCCTGCAGGTCGCC TTGAAGAGCTAAAAATTCTT
CJ1482C ISA F1 F	GAGCTCGGTACCCGGGGATCCTCTAGAGTCGAA ACTAAAAAAAGTTTTTGGCC
CJ1482C ISA F1 R	AAGCTGTCAAACATGAGAACCAAGGAGAATTGA AGAGCTAAAAATTCTTAATTTCA
CJ1482C ISA F2 F	GAATTGTTTTAGTACCTAGCCAAGGTGTGCATA AACTCATTTATAAAAAGGAATTAAAATG
CJ1482C ISA F2 R	AGAATACTCAAGCTTGCATGCCTGCAGGTCCAA GCTCGTTAAAAAAATTTTCTT
CJ1161C ISA F1 F	GAGCTCGGTACCCGGGGATCCTCTAGAGTCAGC TATTGTATTATCAATCTTGCTTTT
CJ1161C ISA F1 R	AAGCTGTCAAACATGAGAACCAAGGAGAATACG CAATTCTTCCATTATAAACG
CJ1161C ISA F2 F	GAATTGTTTTAGTACCTAGCCAAGGTGTGCAAG AATTTAAAAATTTAATTTTTA
CJ1161C ISA F2 R	AGAATACTCAAGCTTGCATGCCTGCAGGTCACA TTCCAAACCATTCTGCAA
CJ1162C ISA F1 F	GAGCTCGGTACCCGGGGATCCTCTAGAGTCCAT GATAATTGTAGCTATTTTGGG
CJ1162C ISA F1 R	AAGCTGTCAAACATGAGAACCAAGGAGAATCAA TTAACATTTTTTACTTTAAATTTCATTTT
CJ1162C ISA F2 F	GAATTGTTTTAGTACCTAGCCAAGGTGTGCTTT ATAATGGAAGAATTGCGTATAAA
CJ1162C ISA F2 R	AGAATACTCAAGCTTGCATGCCTGCAGGTCCTT CTTCTTTAAAAATTTGCAAATACA
CJ1163C ISA F1 F	GAGCTCGGTACCCGGGGATCCTCTAGAGTCTTT GGCTCTAGCTATAGGAGTGAG
CJ1163C ISA F1 R	AAGCTGTCAAACATGAGAACCAAGGAGAATATC TTATATCCTTTTTTGCTTGACAT
CJ1163C ISA F2 F	GAATTGTTTTAGTACCTAGCCAAGGTGTGCAAG GAGTGAAAATGAAATTTAAAGTAA
CJ1163C ISA F2 R	AGAATACTCAAGCTTGCATGCCTGCAGGTCAAA CATTTCAAAATACATAATAATCACACT
CJ1164C ISA F1 F	GAGCTCGGTACCCGGGGATCCTCTAGAGTCGAT TTGGCTTTGCTTATGCTT
CJ1164C ISA F1 R	AAGCTGTCAAACATGAGAACCAAGGAGAATAAT CTACATTACAAACTGGACATAACA
CJ1164C ISA F2 F	GAATTGTTTTAGTACCTAGCCAAGGTGTGCAGC TTTTTGACTTTTAATTTTTATAAAAAT
CJ1164C ISA F2 R	AGAATACTCAAGCTTGCATGCCTGCAGGTCGGT TTTAGCGTCAATTTCTTTT
CJ1165C ISA F1 F	GAGCTCGGTACCCGGGGATCCTCTAGAGTCGTG CTTTAAGCTGGGCTATATATG
CJ1165C ISA F1 R	AAGCTGTCAAACATGAGAACCAAGGAGAATTAA AATAAATTCAATCATCTTAAAATCCC
CJ1165C ISA F2 F	GAATTGTTTTAGTACCTAGCCAAGGTGTGCACT AGACATGCAAAAAAACATTAAAA
CJ1165C ISA F2 R	AGAATACTCAAGCTTGCATGCCTGCAGGTCTGG GAATGATGATGTTCATG
CJ1166C ISA F1 F	GAGCTCGGTACCCGGGGATCCTCTAGAGTCGAA CTATCCAAATTTGTTCCACTAC
CJ1166C ISA F1 R	AAGCTGTCAAACATGAGAACCAAGGAGAATTAT ATCAGGTTTTTCCATATTTTAATAATCT
CJ1166C ISA F2 F	GAATTGTTTTAGTACCTAGCCAAGGTGTGCTTT TGGGATTTTAAGATGATTGA
CJ1166C ISA F2 R	AGAATACTCAAGCTTGCATGCCTGCAGGTCGTT TTAATGTTTTTTTGCATGTCTAG
***Campylobacter jejuni* NCTC 11168 mutant screening primers 5′–3′**	
Cj0908 F screening	TTCCTTGTACTTTATCAAGTAAATTTGG
Cj0911 F screening	AAGCACAGAGCTTAAATCTGG
Cj0911 R screening	ACACTCTTTGGCGCTAGGTT
Cj1154c F screening	CAAGAAAAGGGTATTGTGGC
Cj1154c R screening	GCAAATAATTTCTAGCCAAAAAA
Cj1155c F screening	CATTGCATCGTATTCGAGTT
Cj1155c R screening	GCAAATAATTTCTAGCCAAAAAA
Cj0369c F screening	TTCTTCGATAATTACAAAAGCTCC
Cj0369c R screening	GGCTAATTATATCTTAATTTTGGTTAATAAAA
Cj1483c F screening	CTTCATATAGGATGAAAAAAATATTACAAG
Cj1483c R screening	AATCAAGTTTTAAAAGATATTCAAGATTAAA
Cj1486c F screening	TTTTTCAAGTATAGGTCAATATAATGAAGA
Cj1486c R screening	ATAGTATCACGATTTGCATTACCTATAG
Cj1485c F screening	TTAAACATGGTTCAAAAGGTATGA
Cj1485c R screening	TTTAGTTTCTAACATTTTTTTTCCTCTT
Cj1484c F screening	TGCTGCTTATGTAGCAAAAGA
Cj1484c R screening	TGCTCAAACAAACTTTATCTAAAAAT
Cj1482c F screening	CTATAGGTAATGCAAATCGTGATACT
Cj1482c R screening	TGAGTAAGACCCAAACAATAACTTC
Cj1161c F screening	TGTCAATATGAAGTCCGCTTT
*cj1161c* R screening	AAAATTAATCATCATTTTTAAGAGAGAT
Cj1162c F screening	GGCTTTTATCAATGCTTTAACCA
*cj1162c* R screening	ATATTTTCTAAAATTCTTTGATAATCTTG
Cj1163c F screening	TATCTTATTTCTTTTATACGCAGTGA
*cj1163c* R screening	TTTTAAAGCCTAAAAAAGCATG
Cj1164c F screening	TTTTGATTTGTTGTATAGCACTTG
*cj1164c* R screening	AAGCGGACTTCATATTGACAT
Cj1165c F screening	ATCATATTACTTTAAATGTTGTAGATATGG
*cj1165c* R screening	AAAGAATTTGAAAGGATTGAATAAAT
Cj1166c F screening	GTGCTGCAAACTGAGAATCTC
*cj1166c* R screening	AACCAAATCTACATTACAAACTGGAC
KanR F	ATTCTCCTTGGTTCTCATGTTTGACAGCTTAT
KanR R	GCACACCTTGGCTAGGTACTAAAACAATTC
***Campylobacter jejuni* NCTC 11168 qRT-PCR primers 5′–3′**	
GyrA RT_F	ATGCTCTTTGCAGTAACCAAAAAA
GyrA RT_R	GGCCGATTTCACGCACTTTA
Cj1161c RT_F	TTATGTGAATTCTAGCGGGG
Cj1161c RT_R	CCCAAAGCTACAAGGGTATT
Cj1162c RT_F	TAGAAGTGGATTTGGAGCAA
Cj1162c RT_R	CGCTCTACAATCTCAAAACC
Cj1163c RT_F	TGGCACTTTTAAGCGATACT
Cj1163c RT_R	TGCACCCTTAAACATCATCA
Cj1164c RT_F	AGTGATAGGAGTGGAGTTGA
Cj1164c RT_R	GCCTAGCCAACTTTCTTTCT
Cj1165c RT_F	GGGATTTGGCTTTGCTTATG
Cj1165c RT_R	CTAGAGCCAAAGTCACAGAA
Cj1166c RT_F	ATGCGTAGGAAGAATAGCTG
Cj1166c RT_R	AAAAGCCGAATTTGCCATAG

*Underlined bases are adaptor regions corresponding to the pGEM3zf vector (ISA F and R primers) or to the kanamycin resistance cassette (ISA KR and KF primers).*

### RT-PCR

The SV Total RNA Isolation System (Promega) was used to extract RNA from growing cells of *C. jejuni* in minimal media with or without added copper. Gene specific qRT-PCR primers (Table 2) were designed using PRIMER 3 software (Untergasser et al., 2007) to amplify 200–300 bp sequences within the gene of interest. The *C. jejuni gyrA* gene was used as the reference. All primers were diluted to 25 μM concentration in nuclease-free water and checked for specificity before use by performing a PCR reaction using *C. jejuni* genomic DNA as template. qRT-PCR reactions (20 μl volumes) were carried out in a MX3005P thermal cycler (Agilent) in a MicroAmp^®^ 96-well optical reaction plate (ABI prism). Reactions were performed using either the Sensifast SYBR Lo-ROX one step kit or Sensifast SYBR Hi-ROX one step kit (Bioline, United Kingdom). Each reaction contained 10 μl Sensifast SYBR 2× buffer (Bioline, United Kingdom), 0.2 μl of each gene specific qRT-PCR primer, 0.2 μl reverse transcriptase (Bioline, United Kingdom), 0.4 μl RNAse inhibitor (Bioline, United Kingdom), 2 μl of matched RNA or DNA template and 7 μl nuclease free water (Bioline, United Kingdom). Each reaction using RNA was replicated in triplicate and reactions using genomic DNA were replicated in duplicate for the standard curve. In the thermal cycler, qRT-PCR reactions were carried out at 45°C for 10 min, 95°C for 2 min, followed by 40 cycles of 95°C for 20 s, 55°C for 30 s and 72°C for 20 s each. Data were collected with the associated MxPRO QPCR software (Agilent) and analyzed using Microsoft EXCEL. A standard curve for each gene was established using a series of *C. jejuni* genomic DNA dilutions to normalize for variation in primer annealing efficiency between different primer pairs. The relative expression levels of the target genes were calculated following the standard curve protocol described in the User Bulletin #2 (ABI Prism 7700 Sequence Detection System, Subject: Relative Quantification of Gene Expression) given by Applied Biosystems. Target gene expression was normalized to *gyrA* expression, which acted as an internal control. No-template reactions were included as negative controls for each primer set being used.

### Haem Blotting

Cells were harvested during stationary phase by centrifuging at 17,700×*g* for 20 min at 4°C. Supernatant was discarded. Cell pellet was resuspended in 10 ml of ice-cold 10 mM HEPES buffer (pH 7.4) by gentle pipetting. The cell suspension was sonicated on ice for 6 × 15 s pulses at a frequency of 16 amplitude microns using a Soniprep 150 ultrasonic disintegrator (SANYO) and cell debris/unbroken cells were removed by centrifugation at 12,470×*g* for 20 min at 4°C. The supernatant was removed and centrifuged at 100,000×*g* at 4°C for 1 h in benchtop ultracentrifuge (Beckman). The pellet was washed by pouring off 10 mM HEPES buffer (pH 7.4) twice without disturbing the pellet, which was then re-suspended in 1 ml 25 mM phosphate buffer (pH 7.4) with gentle pipetting, transferred to a glass homogeniser and homogenised gently. Homogenised total membrane protein solution was stored at –80°C and concentration was measured by Lowry assay.

Haem blotting was performed with total membrane proteins according to Vargas et al. (1993) with some modifications. Samples were prepared in sample buffer (60 mM *Tris*–HCl pH 6.8, 2% [w/v] SDS, 0.005% [w/v] bromophenol blue, and 10% [w/v] glycerol) and mildly denatured at 37°C for 30–60 min. Proteins were separated by two separate SDS-PAGE gels. One was stained with Coomassie blue G250 and other was electroblotted onto nitrocellulose membrane (Hybond-C extra, GE Healthcare). SDS-PAGE and nitrocellulose membrane sandwich was made according to manufacturer’s instructions and electroblotting was performed using Mini-Blot Electrophoretic Cell (Bio-Rad) in ice-cold transfer buffer (25 mM *Tris*, 190 mM glycine, 10% [v/v] methanol) for 1 h at constant 100 V. Then the membrane was washed with 20 mM Phosphate buffer for 5 min at RT to remove any residual SDS or methanol and covalently bound haem was detected as haem-associated peroxidase activity (Feissner et al., 2003), using the enhanced chemiluminesence (ECL) kit from GE Healthcare according to manufacturer’s instructions. Membrane was covered with pre-mixed solutions A and B, incubated for 1 min at RT and developed by exposing for different times, as necessary, in ChemiDoc Imaging System (Bio-Rad).

### Membrane Protein Isolation for Blue Native PAGE

Cells were harvested by centrifugation at 17,700×*g* for 20 min at 4°C. Supernatant was discarded and pellet was resuspended in 20 mM *Tris*–HCl pH 7.4, 50 mM NaCl, 1 mM ethylenediaminetetraacetic acid, 10% v/v glycerol, 2 mM dithiothreitol and 0.005% phenylmethylsulfonyl fluoride. Cells were lysed by two passes through a French press (20,000 psi) and cell debris were removed by centrifugation at 12,470×*g* for 20 min at 4°C. Soluble material was loaded onto 40/15% w/w sucrose step gradients and centrifuged at 107,400×*g* in a Beckman SW32 Ti for 10 h at 4°C. Membranes were harvested and protein concentration was measured using the BioRad DC protein assay.

### BN-PAGE

Membranes containing 10 mg ml^–1^ protein were incubated in 2% (w/v) α-dodecylmaltoside (DDM) for 1 h at room temperature. Solubilized protein complexes were isolated and supplemented with a one-tenth volume of blue native sample buffer (100 mM Bis*Tris*–HCl pH 7.0, 0.5 M amino-*n*-caproic acid, 30% (w/v) sucrose, 50 mg ml^–1^ Coomassie Blue G250) and centrifuged for 20 min at 18,000×*g* at 4°C. Sample was loaded onto NativePAGE Bis*Tris* 4–16% gels (Novex) and run at 160 V for 2 h at 4°C in NativePAGE Running Buffer. The cathode chamber was supplemented with 2 ml of 50 mM Tricine, 15 mM Bis*Tris*–HCl pH 7.0, 0.01% Coomassie Blue G250. Imaging was performed using an Amersham Imager 600 in colour.

### Preparation of Peptides by In-Gel Tryptic Digestion and Analysis by Nano-Flow LC-MS/MS

Bands of interest were excised from the BN PAGE and subjected to trypsin digestion according to Pandey et al. (2000). Peptides dissolved in 0.5% (v/v) trifluoroacetic acid (TFA), 3% (v/v) acetonitrile were resolved in triplicate on an EASY-Spray PepMap RSLC C_18_ column (Thermo Scientific, 50 cm × 75 μm ID, 2 μm, 40°C). The gradient profile was delivered at 300 nl min^–1^ by a Dionex RSLCnano chromatography system (Thermo Scientific) as follows: 97% solvent A (0.1% formic acid in water) to 10% solvent B (0.08% formic acid in 80% acetonitrile) over 5 min, then 10–50% solvent B over 30 min. The mass spectrometer was a Q Exactive HF hybrid quadrupole-Orbitrap system (Thermo Scientific) programmed for data dependent acquisition with profile full MS scans at 120,000 resolution and a maximum of ten centroid product ion scans at 30,000 resolution per cycle. Proteins were identified using MaxQuant v. 1.6.10.43 (Cox and Mann, 2008) by searching the MS data files against the UniProtKB reference proteome database for *C. jejuni*^1^ (downloaded on 6 February 2020). Search parameters were: carbamidomethyl-Cys (fixed modification), Met oxidation, protein N-terminal acetylation and Gln to pyro-Glu conversion (variable modifications) with a maximum of two missed cleavages. Proteins were quantified by intensity based absolute quantification (iBAQ; Schwanhäusser et al., 2011) and normalised between MS experiments to total iBAQ.

## Results

### Characterisation of Cytochrome *c* Oxidase in Cells of *C. jejuni*: Cco Activity Is Growth Phase Dependent, Sulphide Sensitive and Not Inhibited by Formate

*Campylobacter jejuni* NCTC 11168 grown under standard microaerobic conditions in complex MHS broth showed high rates of Cco activity (measured as ascorbate/TMPD driven oxygen consumption) of ∼300–400 nmol oxygen consumed min^–1^ mg cell protein^–1^ during the exponential phase of growth. However, the specific activity decreased rapidly as the cells entered stationary phase (Figure 1B). Thus, in all subsequent experiments, cells were harvested at mid-exponential phase to ensure maximal activity and valid comparisons between wild-type and mutant strains. Previously constructed deletion mutants in either the *ccoNOPQ* genes or *cioAB* genes encoding the major subunits of the two oxidases in *C. jejuni* (Liu and Kelly, 2015), were used as controls in subsequent experiments and to characterise the response of each oxidase to exogenous sulphide. With formate as a physiological electron donor, oxygen consumption by the *cioAB* mutant, where Cco is the only active oxidase, was severely inhibited by sulphide, whereas respiration in the *ccoNOQP* mutant was sulphide resistant up to 1 mM (Figure 1C). This agrees with studies in other bacteria where *bd*-type quinol oxidases are much more sulphide resistant than HCO’s (Forte et al., 2016; Korshunov et al., 2016) and also correlates with the previously documented cyanide resistance of the *C. jejuni* quinol oxidase (Jackson et al., 2007). Finally, it has been reported that formate stimulates growth but actually inhibits the Cco activity in *C. jejuni* (Kassem et al., 2017). We found that addition of formate to cell suspensions in an oxygen electrode in the presence of ascorbate/TMPD, stimulated oxygen consumption compared to ascorbate/TMPD alone (Figure 1D). A stimulation was also seen in the *ccoNOQP* operon deletion mutant, where formate derived electrons reduce oxygen via the alternative quinol oxidase CioAB. Interestingly, stimulation was also observed in the *cioAB* deletion mutant, which is unexpected if we assume the Cco complex is operating at maximal rate with excess ascorbate/TMPD. Using higher concentrations of ascorbate or TMPD did not alter these rates, so the stimulation may be due to an unidentified oxidase independent oxygen consuming process. We conclude that formate does not inhibit oxidase activity.

### Deletion of *ccoI* or *ccoS* Homologues Abolishes Cco Activity, Which Is Partially Rescued by Excess Copper

**FIGURE 2:**
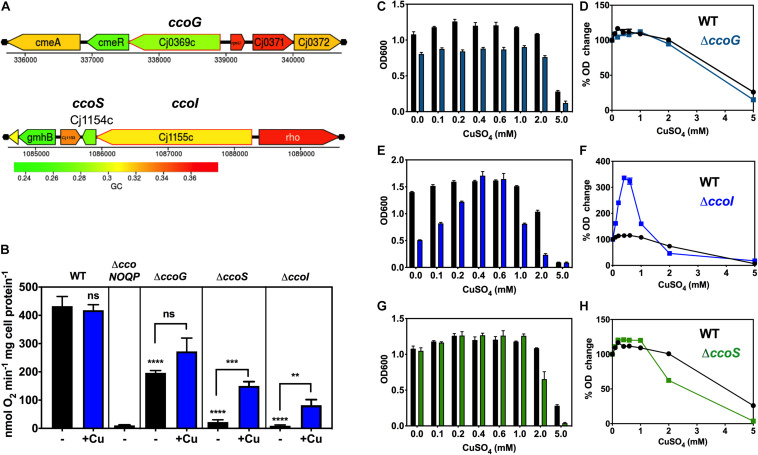
Dependence of oxidase activity and growth on putative *ccoG*, *ccoI*, and *ccoS* genes and the effect of exogenous copper. **(A)** Genomic regions of NCTC 11168 encoding putative *ccoG* (*upper panel*), *ccoI* and *ccoS* (*lower panel*) genes. Images derived from campyDB (http://xbase.warwick.ac.uk/campydb/) and genes coloured by GC composition (see scale). **(B)** Cytochrome *c* oxidase activity measured with ascorbate/TMPD as electron donor. Wildtype, Δ*ccoNOQP*, Δ*ccoG*, Δ*ccoI*, and Δ*ccoS* cells were grown in unsupplemented Mueller-Hinton medium (approx. 0.05 μM copper) or with copper sulphate added to 0.6 mM (an excess but non-toxic level; see Figure 3). The data are the average of triplicate cultures with error bars showing SD. *t*-tests were performed to test significance (*p* > 0.05, ns; 0.01 > **p > 0.001; 0.001 > ***p > 0.0001; ****p > 0.0001). The stars above the black bars represent the statistical significance of comparisons between the unsupplemented mutant and wild-type rates. Panels **(C–H)**, copper sensitivity of growth of wild-type and *ccoG*, *ccoI*, and *ccoS* mutants. WT, Δ*ccoG* **(C)**; Δ*ccoI* **(E)**; and Δ*ccoS* **(G)** cells were grown at different concentrations of CuSO_4_ in Mueller-Hinton medium until stationary phase (24 h) and the final ODs were measured. The data are the average of triplicate cultures with error bars showing SD. In panels **(D,F,H)**, the corresponding raw data for each strain are normalised with respect to the final OD without Cu = 100%, to better visualise the degree of enhancement or inhibition of growth by copper.

In *C. jejuni* NCTC 11168, the genes *cj1490c*, *cj1489c*, *cj1488c*, and *cj1487c* encode the known CcoNOQP subunits, respectively, of the *cbb_3_-*type cytochrome *c* oxidase (Liu and Kelly, 2015), but the genes required for copper trafficking to the periplasm and insertion of copper into CcoN have not been identified. In many bacteria, a *ccoGHIS* operon is situated downstream of *ccoNOQP*, but in *C. jejuni*, candidate *ccoGHIS* genes are scattered throughout the genome and have not been functionally characterised previously. Genome annotation and BLAST searches showed that *cj0369* encodes a CcoG homologue, *cj1154c* encodes a CcoS homologue and *cj1155c* encodes a CcoI homologue, which may have a copper translocating function (Figure 2A). Kanamycin resistant deletion mutants of the cognate genes were made by replacing most of the coding regions with a kanamycin resistance cassette via homologous recombination (See “Materials and Methods”). This cassette carries a constitutive promoter and no terminator. Its insertion has been shown to result in expression of operonic downstream genes (Guccione et al., 2008, 2010) thus minimizing polar effects. The deletion mutants formed by transformation of *C. jejuni* cells with recombinant plasmids were checked for correct mutation by colony PCR with a combination of screening and kanamycin cassette primers (Table 2).

Figure 2B shows from ascorbate/TMPD driven oxygen consumption rates of wild-type and mutants strains that in the *ccoG* (*cj0369c*) deletion mutant, Cco activity was reduced by ∼50%. There was an indication of a stimulation of activity in the mutant by adding excess Cu, but this just failed to reach statistical significance (*P* = 0.0524). In contrast, in the *cj1155c* and *cj1154c* deletion mutants, lacking the *ccoI* and *ccoS* homologues, respectively, Cco activity was almost abolished but was rescued after growth with excess copper to similar extents (6.7-fold increase in activity for *ccoS* and 9.1-fold for *ccoI* mutants) (Figure 2B). These data are consistent with the involvement of CcoI and CcoS in copper supply to CcoNOQP.

### Deletion of *ccoI* Causes a Severe Growth Defect That Is Fully Rescued by Excess Copper

Copper sensitivity of growth assays were performed with wild-type, Δ*ccoS* (*cj1154c*), Δ*ccoI* (*cj1155c*), and Δ*ccoG* (*cj0369c*) strains in complex MHS media by measuring final cell densities in triplicate cultures. ICP-MS analysis showed that the copper content of MHS was ∼0.05 μM; a small stimulation in growth on adding up to 0.2 mM of CuSO_4_ to both wild-type and mutant strains was noted. More than 2 mM CuSO_4_ was toxic for the bacterial cells but up to ∼1 mM had no significant toxicity in these media (Figures 2C–H).

The *ccoG* mutant showed a small growth defect that was insensitive to varying copper concentration compared to WT (Figures 2C,D). In contrast, cells lacking the CcoI homologue (Cj1155) were found to have a significant growth defect and were highly responsive to the external Cu concentration (Figures 2E,F). Increases in CuSO_4_ concentration initially strongly stimulated growth and 0.4 mM completely complemented the absence of the *cj1155c* gene product. However, above 0.6 mM growth was inhibited more than the wild-type. Cells lacking the CcoS homologue (Cj1154) did not have a growth defect but growth was inhibited more than the wild-type at CuSO_4_ concentrations above 1 mM (Figures 2G,H).

### Role of the *cj0908–0911* Operon in Oxidase Assembly

**FIGURE 3:**
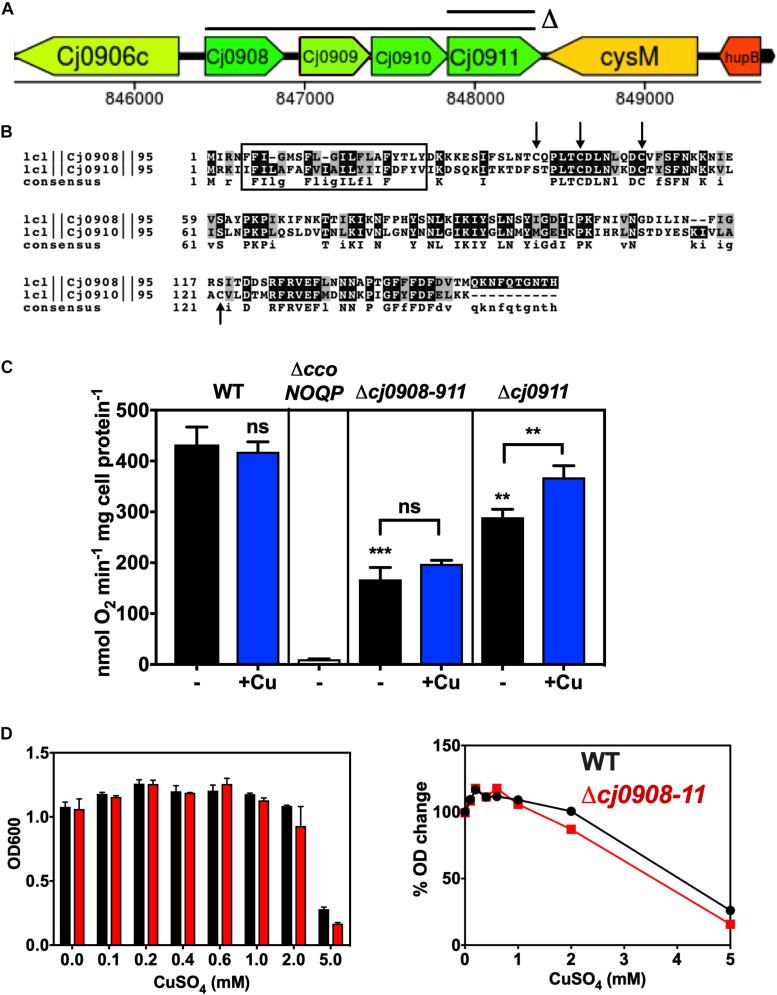
Role of the *cj0908–911* genes in Cco activity. **(A)** Organisation of the genomic region surrounding *cj0908–911* and the extent of deletions made. An operon deletion plasmid in the pGEM3Zf vector was made using primers Cj0908 ISA F1 F, Cj0908 ISA F1 KR, Cj0911 ISA F2 KF and Cj0911 ISA F2 R (Table 2) to create the flanks between which the Kan cassette was inserted. Image derived from campyDB (http://xbase.warwick.ac.uk/campydb/) and genes coloured by GC composition as in the legend to Figure 2. **(B)** Alignment of Cj0908 and Cj0910 using T-COFFEE and BOXSHADE to show identical residues (Black boxes) and conservative substitutions (Grey boxes). The rectangular boxed area shows the single transmembrane helix predicted using TMHMM. Arrows highlight Cys residues in either or both proteins, which may have a redox function or bind Fe. **(C)** Cytochrome c oxidase activity measured with ascorbate/TMPD as electron donor. Wildtype, Δ*ccoNOQP*, Δ*cj0908-cj0911*, and Δ*cj0911* cells were grown in Mueller-Hinton medium without any added copper or with copper sulphate added to 0.6 mM. The data are the average of triplicate cultures with error bars showing SD. Students *t*-tests were performed to check significance (*p* > 0.05, ns; 0.01 > **p > 0.001; 0.001 > ***p > 0.0001). The stars above the black bars represent the statistical significance of comparisons between the unsupplemented mutant and wild-type rates. **(D)** Copper sensitivity of wild-type and *cj0908–911* mutant determined in the same way as described in the legend to Figure 3. *Left panel*; raw final cell density data; *Right panel*, data normalised to cell yield without copper = 100%.

We hypothesised that the operonic genes *cj0908–911* (Figure 3A) may encode proteins needed to transfer Cu from the periplasm to the CcoN subunit, as Cj0909 is a putative periplasmic Cu(I) binding protein homologous to bacterial PCu_*A*_C and Cj0911 is a protein belonging to the Sco1 family. As described above, members of this family are required in other bacteria as a Cu chaperone for the proper assembly of cytochrome *c* oxidase. However, unlike Cj0909 and Cj0911, Cj0908 and Cj0910 are paralogous proteins (41% identical) that have no characterised counterparts in Cco assembly systems in other bacteria. Interestingly, Cj0908 has a cysteine motif similar in part to thioredoxin−like [2Fe–2S] ferredoxins (Trx−like−Fd’s) of C–X_4_–C–X_6_–C, while Cj0910 is missing the first Cys but has the C–X_6_–C and an additional Cys at position 122 (Figure 3B). The SignalP and TMHMM servers predict no signal sequence but a single N-terminal transmembrane helix at the N-terminus of each protein, with the remainder of the protein likely to be periplasmic (Figure 3B).

A deletion mutant was constructed that removed the entire *cj0908–911* operon, which was replaced with a kanamycin cassette. In addition, an individual *cj0911* deletion mutant was made (Figure 3A). The data in Figure 3C shows that both of these mutants were clearly affected in cytochrome c oxidase activity. In the Δ*cj0908–cj0911* strain activity was significantly reduced (over 50% lower) but was not abolished, suggesting that these gene products are involved in CcO assembly but are not essential for it. In the Δ*cj0911* mutant, lacking the Sco1 homologue, activity was also reduced but to a lesser extent compared to the entire operon mutant. A significant stimulation in activity on adding excess Cu is consistent with the involvement of Cj0911 in handling Cu, although this would need to be demonstrated biochemically. In contrast the operon mutant did not show stimulation of oxidase activity by copper (Figure 3C). In addition, growth experiments (Figure 3D) showed that the Δ*cj0908–cj0911* operon mutant was insensitive to varying amounts of Cu with respect to WT. Additional studies with individual mutants and analysis of the gene products will be needed to fully define the roles of *cj0908* and *cj0910*.

### Deletion of the *cj0908–11* Operon, *cj1154c* or *cj1155c* Does Not Affect Biogenesis of the CcoO Subunit of Cytochrome *c* Oxidase

**FIGURE 4:**
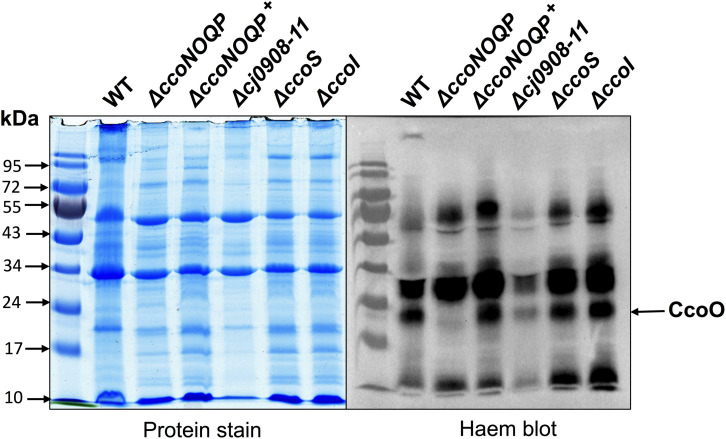
Deletion of *cj0908–911*, *ccoS* or *ccoI* does not affect insertion of the CcoO cytochrome *c* into the cytoplasmic membrane. Samples of total membrane proteins of wild-type, Δ*ccoNOQP*, Δ*ccoNOQP*^+^, Δ*cj0908-cj0911*, Δ*ccoS*, and Δ*ccoI*, were mildly denatured in the absence of mercaptoethanol to preserve attachment of C-haems to Cys residues and 20 μg protein in each lane was loaded on 10% SDS-PAGE gels. Gels were either stained with Coommassie brilliant blue (*left panel*) or for haem-associated peroxidase activity (*right panel*) using a standard enhanced chemiluminescence kit (See section “Materials and Methods”). The CcoO band (arrowed) is missing in Δ*ccoNOQP* only. CcoP is not visible because of intense c-type cytochrome bands of a similar size.

Total membrane preparations of wild-type, Δ*ccoNOQP*, Δ*ccoNOQP^+^*, Δ*cj0908-cj0911*, Δ*ccoS*, and Δ*ccoI* cells were made as described in “Materials and Methods” and gently denatured in the absence of mercaptoethanol to preserve haem binding to *c*-type cytochromes. Figure 4 shows the membrane cytochrome profiles after SDS-PAGE and protein blotting, with detection by haem-associated peroxidase activity. The two cytochrome *c* proteins associated with the oxidase complex, CcoO and CcoP have molecular masses of ∼25 and ∼31 kDa, respectively. CcoP could not be identified on these gels as it is about the same size as a very abundant cytochrome of a similar size but a band corresponding to the approximate size of CcoO is clearly absent in the *ccoNOQP* deletion mutant but present in both wild-type and complemented strains. This band is also present in membranes of the biogenesis mutants Δ*cj908–911*, Δ*ccoS*, and Δ*ccoI*. This suggests that CcoO can still be inserted into the membrane in the absence of biogenesis proteins that are needed for CcoN assembly (i.e., copper insertion).

### Role of the *ccoH* Homologue *cj1483c* and Identification of *ccoX*, *ccoY*, and *ccoZ*; Novel Genes Downstream of *ccoP* That Are Required for Normal Oxidase Activity

**FIGURE 5:**
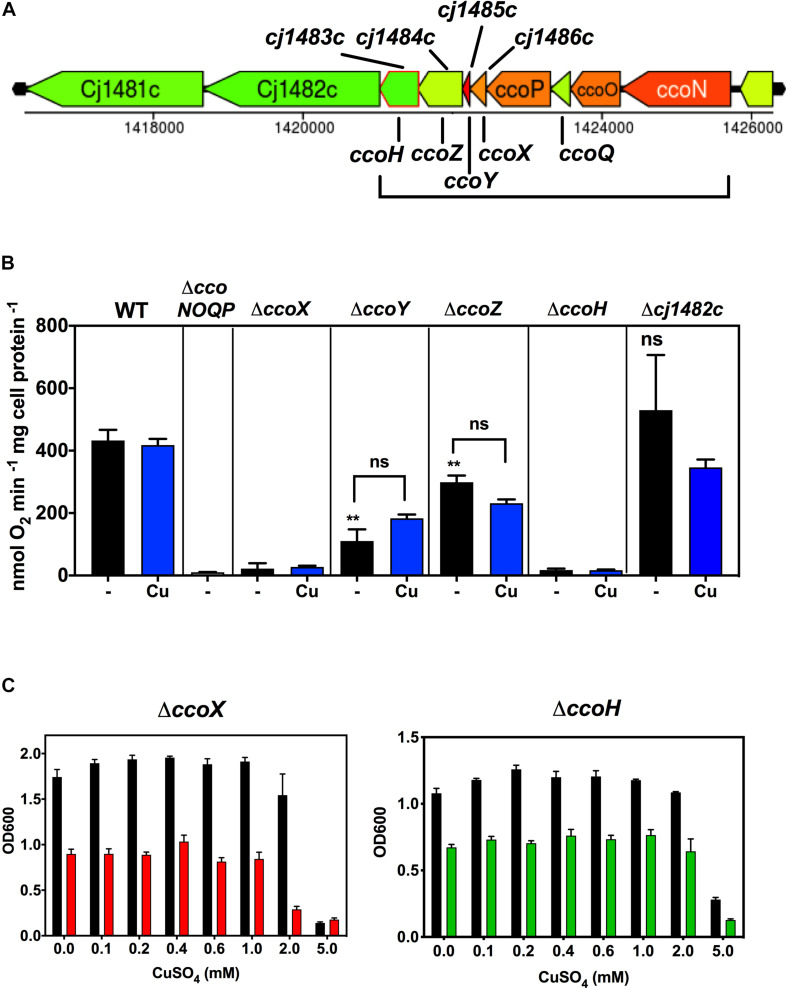
Genes downstream of *ccoP* are required for Cco activity. **(A)** Gene organisation at the *ccoNOQP* locus. Image derived from campyDB (http://xbase.warwick.ac.uk/campydb/) and genes coloured by GC composition as in the legend to Figure 2. **(B)** Wildtype, Δ*ccoNOQP*, Δ*ccoX* (*cj1486c*), Δ*ccoY* (*cj1485c*), Δ*ccoZ* (*cj1484c*), Δ*ccoH* (*cj1483c*), and Δ*cj1482c* cells were grown in unsupplemented Mueller-Hinton medium or with copper sulphate added to 0.6 mM, as indicated. Cytochrome *c* oxidase activity was measured with ascorbate/TMPD as electron donor. Students *t*-tests were performed to check significance (*p* > 0.05, ns; 0.01 > **p > 0.001). The stars above the black bars represent the statistical significance of comparisons between the unsupplemented mutant and wild-type rates. **(C)** Growth and copper sensitivity profiles of the Δ*ccoX* and Δ*ccoH* strains. In panels **(B,C)**, the data are the average of triplicate cultures with error bars showing SD.

*cj1483c* encodes a CcoH homologue, which in other bacteria is thought to be tightly associated with the oxidase. Intriguingly, although this *ccoH* homologue is just downstream of the structural gene *ccoP* (*cj1487c*), three intervening genes of unknown function (*cj1484c*, *cj1485c*, and *cj1486c*) are also present at this locus (Figure 5A). Given this gene organisation we constructed individual deletion mutants in each of these genes, in addition to a *cj1482c* mutant, to assess their Cco activity in comparison to isogenic wild-type and *ccoNOQP* deletion strains (Figure 5B). In both the *ccoH* (*cj1483c*) and *cj1486c* deletion mutants, oxidase activity was abolished and was not restored by adding excess copper, suggesting an absolutely essential role in the activity of the oxidase (Figure 5B). Both of these mutants showed a microaerobic growth defect (reduction in final cell density) and did not show any copper stimulation of growth compared to the wild-type (Figure 5C). Cj1486 is predicted by the TMHMM server to have a single transmembrane helix and is a member of a conserved family of small proteins of unknown function containing the DUF4006 domain. In the *cj1484c* and *cj1485c* mutants, in contrast to *cj1486c*, Cco activity was significantly decreased but not abolished. This phenotype could not be complemented by addition of exogenous copper (Figure 5B). Cj1485 consists of just 33 residues and is predicted by the TMHMM server to have a central transmembrane helix. Cj1484 (200 residues) contains a DUF5130 domain and a TPM_phosphatase domain. Finally, deletion of *cj1482c* had no effect on Cco activity compared to the wild-type, so we conclude that this gene is not involved in oxidase activity or biogenesis. In view of the abolished or compromised oxidase phenotypes of the *cj1486c*, *cj1485c*, and *cj1484c* mutants and the genomic linkage to both *ccoH* and *ccoNOQP*, we conclude that the proteins encoded by these genes are crucial for normal oxidase activity. We therefore designate these genes *ccoX* (*cj1486c*), *ccoY* (*cj1485c*), and *ccoZ* (*cj1484c*).

### CcoH and CcoZ Are Associated With Oxidase and Cytochrome *bc* Complex Proteins in a High Molecular Weight Band After Detergent Solubilisation of Membranes

**FIGURE 6:**
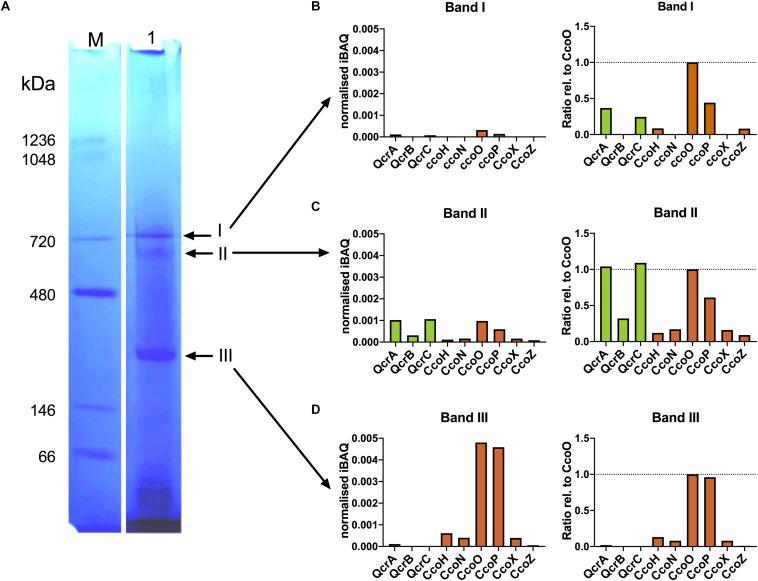
Mass spectrometry reveals co-migration of putative Qcr-oxidase supercomplex components in blue-native PAGE. **(A)** BN-PAGE analysis of solubilised membrane fractions from *C. jejuni*. Membranes solubilised with 2% (w/v) αDDM were subjected to BN-PAGE separation as described in “Materials and Methods” (*Lane 1*). Arrows indicate bands (I–III) excised and subjected to in-gel tryptic digestion and analysed by mass spectrometry. *Lane M* contains molecular size markers. **(B–D)** Mass spectrometry-based quantification of Qcr (green) and Cco (brown) proteins by median iBAQ (*left panels*), and protein abundance ratios relative to CcoO (*right panels*), for excised gel bands I-III.

Modelling using the Phyre^2^ server (Kelley et al., 2015) showed that CcoZ (Cj1484) is structurally similar to Msmeg_4692, a protein that is part of an oxidase-cytochrome *bcc* supercomplex in the actinobacterium *Mycobacterium smegmatis* (Gong et al., 2018; Wiseman et al., 2018). Comparison of Cj1484 to Msmeg_4692 as the template (Supplementary Figure 1) shows the same mixed beta sheet/alpha helical fold with a TPM domain found in some phosphatases present in both proteins. This raised the possibility that such a supercomplex might exist in *C. jejuni*. The cytochrome *bc* complex (menaquinol:cytochrome c reductase) in *C. jejuni* is encoded by the *qcrABC* genes (preferred designation although annotated as *petABC*) and contains a dihaem cytochrome *c*_4_ (QcrC) in place of cytochrome c_1_ (Garg et al., 2018). We solubilised membrane preparations of wild-type cells with the detergent DDM and separated proteins by BN-PAGE (Figure 6A), which revealed the presence of several high molecular weight bands, the most prominent of which were designated band I (apparent molecular weight ∼730 kDa), band II (apparent molecular weight ∼600 kDa) and band III (apparent molecular weight ∼200 kDa). These bands were excised for trypsin digestion and the resultant peptides analysed by nanoflow LC-MS/MS as described in “Materials and Methods”. The proteins contained in the bands were identified using the MS data-files as input for database searching and the complete results are shown in Supplementary Table 2. The number of proteins identified in bands I, II and III was 403, 622, and 717, respectively, and, in all cases, the highest molecular mass represented was 168.8 kDa. Therefore, the bands contain populations of proteins co-migrating in both specific and non-specific complexes that are stable in the presence of DDM. The MS analysis identified nine protein components of the putative Qcr-oxidase supercomplex: CcoN, CcoO, CcoP, CcoH (Cj1483), CcoZ (Cj1484), CcoX (Cj1486), QcrA, QcrB, and QcrC. A quantitative comparison of these proteins, using mass spectral peak intensities normalised to approximate molar amount, revealed that band I contained very low levels of CcoO, CcoP, CcoH, CcoZ, QcrA, and QcrC (Figure 6B). Band II contained all nine proteins and, for the proteins also identified in band I, their quantification indicates an abundance of around 10-fold higher than in band I (Figure 6C, left panel). Expression of protein abundance relative to CcoO suggests a stoichiometry of 1:1:1 for CcoO, QcrA, and QcrC (Figure 6C, right panel). This correlation may reflect the occurrence of a complex *in vivo* that comprises both Cco and Qcr subunits. The sub-stoichiometric amounts of some subunits would therefore probably result from co-migrating populations of partially assembled precursor sub-complexes of a putative Qcr-oxidase complex, or from possible concealment of trypsin cleavage sites in transmembrane regions resulting in underrepresentation of those tryptic peptides in the mass spectra. The possible existence of intermediate sub-complexes in the formation of a functional Qcr-oxidase complex is supported by the quantification of CcoO and CcoP in a 1:1 ratio in band III (Figure 6D). While CcoH and CcoX were also quantified in band III at a similar stoichiometric ratio to band II, CcoZ was depleted 10-fold. This depletion was associated with an absence of QcrB and QcrC, and a substantial reduction in QcrA. Since absolute stoichiometry determinations by label-free MS may be indicative rather than decisive, particularly for membrane proteins where MS intensities may be underrepresented, further work will be needed to confirm the existence of a Qcr-oxidase complex and elucidate the mechanism of its assembly.

### Characterisation of a Metal Homeostasis Gene Cluster and Relationships Between Copper Homeostasis and Cytochrome *c* Oxidase Activity

**FIGURE 7:**
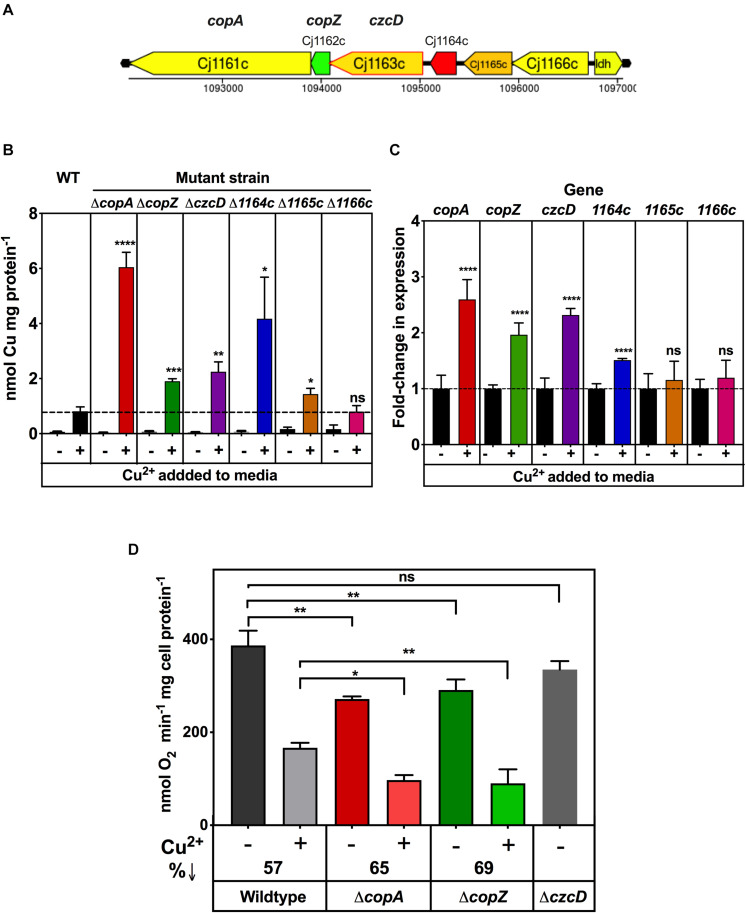
Impairment of copper homeostasis negatively affects Cco activity. **(A)** Arrangement of genes encoding potential metal homeostasis proteins in *C. jejuni*. Genes are colour coded according GC content as in the legend to Figure 2. **(B)** Intracellular Cu accumulation in wild type, Δ*copA*, Δ*copZ*, Δ*czcD*, Δ*cj1164c*, Δ*cj1165c*, and Δ*cj1166c* cells grown either in unsupplemented MEMα minimal medium or with 0.25 mM copper sulphate added. Intracellular Cu content at stationary phase was measured by ICP-MS and normalised to total cell protein concentration as measured by Lowry assay. The data are the means of independent triplicate cultures with error bars showing SD. One-way ANOVA was performed to determine the significance of the difference between wild-type plus copper and the corresponding mutants plus copper (*p* > 0.05, ns; 0.05 > *p > 0.01; 0.01 > **p > 0.001; 0.001 > ***p > 0.0001; 0.0001 > ****p). **(C)** Effect of copper on gene expression, measured by qRT-PCR. *C. jejuni* wildtype cells were grown with or without 0.25 mM copper sulphate as in panel **(B)**. RNA was extracted (see “Materials and Methods”) and qRT-PCR performed using primers shown in Table 2 with the *gyrA* gene used as an internal control. The data are the means of independent triplicate cultures with error bars showing SD. For each gene, Students *t*-test was performed to determine the significance of the difference between expression measured without added copper (set to onefold in each case) and with added copper (*p* > 0.05, ns; 0.0001 > ****p). **(D)** Cytochrome *c* oxidase activity in copper homeostasis mutants. *C. jejuni* wildtype, Δ*copA*, Δ*copZc*, and Δ*czcD* cells were grown in 5 ml MEMα minimal media in six-well plates, three wells with 0.25 mM copper sulphate and the other three wells without. Cytochrome *c* oxidase activity was measured by ascorbate/TMPD driven oxygen uptake normalised to total cell protein. The data are the average of independent triplicate cultures with error bars showing SD. One-way ANOVA was performed to determine the significance of the difference between wildtype and the mutants shown, either without or with added copper sulphate (*p* > 0.05, ns; 0.05 > *p > 0.01; 0.01 > **p > 0.001). The decrease in oxidase activity caused by growth with excess copper is indicated as%↓.

Cj1161 is homologous to bacterial CopA proteins (Hall et al., 2008), known to be P-type ATPases that have a copper efflux function related to maintaining intracellular Cu homeostasis (Rensing et al., 2000). Indeed, a previously constructed *cj1161c* mutant showed increased copper sensitivity consistent with this role (Hall et al., 2008). However, the *cj1161c* gene is encoded in a cluster (*cj1161c–cj1166c*) that may have related or additional functions in copper or metal homeostasis (Figure 7A). Cj1162 is a CopZ homologue; CopZ is a cytoplasmic copper chaperone that transfers copper to CopA (Singleton and Le Brun, 2007). Cj1163 is annotated as a Zn-Co-Ni P-type ATPase transporter (a CzcD homologue) and has recently been shown to be a thermoregulated Zn exporter (Barnawi et al., 2020). Cj1164 is homologous to Zn finger proteins. Cj1165 and Cj1166 are predicted to be integral membrane proteins with six transmembrane helices. They both have sequence similarity to serine/threonine efflux proteins, suggesting that they are not relevant for metal homeostasis. In order to assess the contribution of this gene cluster to Cco activity, deletion mutants were made in each of the *Cj1161c–cj1166c* genes individually and initially assayed for copper sensitivity, intracellular Cu content and the effect of Cu on gene expression.

Supplementary Figure 2 shows copper sensitivity profiles for these mutants in minimal media (based on MEM-alpha where the unsupplemented copper concentration determined by ICP-MS was ∼0.02 μM). Exogenously added CuSO_4_ stimulated growth up to a concentration of 0.1 mM in the wild type and all of the mutants, but Δ*copA*, Δ*copZ*, Δ*czcD*, and Δ*cj1164c* strains were significantly more sensitive to Cu than the wild type above this concentration. For each of these mutants, the profiles were broadly similar, with a concentration of ∼0.25 mM CuSO_4_ resulting in a ∼50% reduction in final cell density compared to the wild type (Supplementary Figure 2). The results also showed that Δ*cj1165c* and Δ*cj1166c* were not differentially sensitive to Cu with respect to wild type (Supplementary Figure 2), consistent with these genes being uninvolved in copper homeostasis. Zinc sensitivity assays were performed on the *czcD* deletion strain (Supplementary Figure 3), which confirmed that it is indeed much more sensitive to Zn compared to the wild type or Δ*copA* strains (Supplementary Figure 3A). Comparison of the intracellular zinc levels in the wild-type and *czcD* mutant by ICP-MS revealed a significant accumulation of zinc in the mutant cells (Supplementary Figure 3B) supporting the results of Barnawi et al. (2020).

Inductively Coupled Plasma Mass Spectrometry was also used to measure total intracellular Cu in wild type and deletion mutant strains after growth in minimal media, with and without 0.25 mM excess CuSO_4_. The results (Figure 7B) showed that when cells were grown in a Cu limiting environment, the intracellular Cu concentrations were very low and there was no significant difference between wild type and any of the mutant strains. However, growth in the presence of 0.25 mM CuSO_4_ resulted in higher intracellular Cu concentrations in Δ*copA* (∼7.5-fold), Δ*copZ* (∼2.4-fold), Δ*czcD* (∼2.8-fold), and Δ*cj1164c* (∼5.2-fold) knock out mutants compared to wild type cells grown with 0.25 mM CuSO_4_. In contrast, Δ*cj1165c* showed only a ∼1.7-fold increase and Δ*cj1166c* showed no increase in intracellular copper compared to the wild-type. Overall, these results correlate well with the pattern of copper sensitivity shown by these mutants (Supplementary Figure 2). In order to examine patterns of copper dependent gene expression, qRT-PCR analysis was carried out on wild-type cells grown to mid-exponential phase in minimal media with or without 0.25 mM CuSO_4_ (i.e., the same conditions used for the ICP-MS analysis above). Figure 7C shows that expression of *copA*, *copZ*, *and czcD* genes was increased ∼2–3 fold by exogenous Cu and *cj1164c* expression increased ∼1.5-fold. The expression of *cj1165c* and *cj1166c* was not increased by copper under these conditions.

From the above results and a consideration of the properties of the encoded proteins, only the *copA* and *copZ* genes are most clearly involved in Cu homeostasis in *C. jejuni*, while Cj1163 (and possibly Cj1164, though this was not further studied here) is involved in zinc homeostasis (Barnawi et al., 2020). Ascorbate/TMPD driven oxygen uptake assays for oxidase activity showed that the *copA* and *copZ* knock out mutants, but not the *czcD* mutant, have significantly lower Cco activity than wild type cells, when grown in the absence of any added copper (Figure 7D). Furthermore, addition of a concentration of CuSO_4_ that is growth inhibitory in the minimal media used (0.25 mM) caused significant inhibition of Cco activity in the wild-type and this effect was exacerbated in both the *copA* and *copZ* mutants (Figure 7D). Overall, the data suggest that disruption in intracellular Cu homeostasis as well as the availability of Cu in the bacterial growth environment affects the activity of the cytochrome *c* oxidase.

## Discussion

The Cco complex is a key proton-motive respiratory component in microaerobically grown *C. jejuni*. Its activity is high in actively growing cells, but declines during stationary phase. The intestine is a sulphide rich environment and we confirmed from the results of our mutant studies the sulphide sensitivity of the *C. jejuni* Cco complex and sulphide resistance of CioAB seen in other bacteria (Forte et al., 2016; Korshunov et al., 2016). However, it has been reported previously that a *ccoN* mutant is unable colonise the chicken host, while *cioAB* mutants were found to colonise normally (Weingarten et al., 2008) which is not consistent with this pattern of inhibition. Perhaps the niche occupied by *C. jejuni* is deep within the mucus layer overlying the intestinal epithelium where there is sufficient oxygen but low enough sulphide to enable continued Cco activity.

A continued supply of copper is clearly essential for Cco assembly and activity, but how copper is imported into *C. jejuni* is unknown. Unlike many bacteria, sequenced strains of *C. jejuni* do not seem to possess the CcoA transporter that has been shown to be important in uptake of copper for oxidase activity in *Rhodobacter* and many other genera (Ekici et al., 2014). In this work, we have therefore focused on identifying those genes that are involved in exporting copper from the cytoplasm to the periplasm for incorporation into the CcoN subunit via periplasmic chaperones.

Recent evidence suggests that the key transporter for copper export across the cytoplasmic membrane for oxidase assembly in Gram-negative bacteria is the P-type ATPase CcoI, assisted by CcoG, which is now known to act as a specific cupric reductase (Marckmann et al., 2019). Unlike some other *cbb_3_-*type Cco containing bacteria, which have a *ccoGHIS* operon adjacent to *ccoNOQP*, we found that potential *ccoG*, *ccoI*, and *ccoS* genes are scattered throughout the genome in *C. jejuni* NCTC11168. Cj0369 is a membrane bound CcoG orthologue. The absence of Cj0369 reduced Cco activity substantially but this protein is clearly not essential for complex activity, suggesting other mechanisms of cupric reduction exist in the cytoplasm. In *R. capsulatus*, deletion of CcoG had more significant effects on the activity of the *cbb*_3_-type Cco (Marckmann et al., 2019). The loss of activity was not rescued by growing cells with excess Cu in the medium suggesting Cj0369 is not involved directly in export, but does not rule out a redox role in copper handling.

Deletion of the *cj1155c* gene encoding a CcoI homologue completely abolished Cco activity but the activity was largely rescued on growing cells with excess copper. The mutant also showed a severe growth defect, which was completely rescued by CuSO_4_ when added up to 0.6 mM, above which the Cu became inhibitory. Although this phenotype is consistent with the expected copper export role for CcoI, the ability of both the growth and oxidase phenotypes to be rescued by copper contrasts with the phenotype of a *ccoI* mutant in *R. capsulatus* (Koch et al., 2000) where exogenous copper does not rescue oxidase activity. The severity of the *ccoI* mutant growth defect in *C. jejuni* is also much greater than that of a *ccoNOQP* deletion mutant, suggesting an additional role. The phenotype is in fact more similar to that reported after deletion of a *ccoI* homologue called *ctpA* in *Rubrivivax gelatinosus* (Hassani et al., 2010). CtpA was shown to have a pleiotropic role in supplying copper not only for the *cbb*_3_-type oxidase in *R. gelatinosus*, but also for the alternative *aa*_3_-oxidase and the periplasmic nitrous oxide reductase, NosZ and the oxidase activity defect of a *ctpA* deletion mutant could be largely rescued by copper (Hassani et al., 2010). However, *C. jejuni* NCTC11168 does not possess these other copper containing respiratory complexes. The only other known periplasmic copper containing proteins in strain NCTC 11168 are the multi-copper oxidase CueO (Cj1516) involved in copper homeostasis (Hall et al., 2008) and the p19 protein, which is part of an iron uptake system (Chan et al., 2010; Liu et al., 2018). We found that CueO dependent phenoloxidase activity was unchanged in the *ccoI* mutant (unpublished observations) but further work will be required to investigate any connection with p19 or to identify other systems which may be dependent on CcoI. In *R. capsulatus*, the CcoNOQP complex was absent in a strain lacking *ccoI*, but CcoP itself was still detectable (Kulajta et al., 2006). However, a more recent study has shown that CcoN can be membrane inserted in the absence of CcoO or CcoP (Ekici et al., 2013), but the absence of CcoN (as would be expected in biogenesis mutants) usually results in loss of both of the c-type cytochromes in the complex. Although we could not visualise CcoP, haem blots did suggest the presence of CcoO in membranes of the *C. jejuni ccoI* mutant, possibly indicating independent assembly of the dihaem cytochrome c from the rest of the complex. This is also different to the situation in the absence of the CcoI homologue CtpA in *R. gelatinosus*, where both CcoO and CcoP were significantly reduced (Hassani et al., 2010). It would clearly be informative to know what the status of CcoN is in our biogenesis mutants but unfortunately we could not detect CcoN on immunoblots using *R. capsulatus* CcoN antibodies.

The Δ*cj1154c* strain lacking the CcoS homologue also completely lacked Cco activity. Koch et al. (2000) reported the necessity of CcoS in *R. capsulatus* for the presence of haem *b*, haem *b*_3_, and Cu_*B*_ cofactors in the CcoN subunit. In *R. capsulatus* cells lacking CcoS, a CcoNOQP complex was detected but inactive (Kulajta et al., 2006). We found a copper sensitive phenotype for the Δ*cj1154c* strain and the addition of excess Cu to the growth medium partly rescued the Cco activity, but in contrast to the *ccoI* mutant, to only ∼20% of that in *C. jejuni* wild-type cells. This nevertheless suggests that Cj1154 might be required for copper handling for oxidase activity.

The periplasmic copper chaperones PCuC and Sco1 act as a relay system for insertion of Cu into CcoN (Trasnea et al., 2018). In *C. jejuni*, we identified Cj0909 as a PCuC homologue and Cj0911 as a Sco1 homologue. *cj0909* and *cj0911* form an operon with *cj0908* and *cj0910*, genes of unknown function. Both Δ*cj0908–cj0911* and Δ*cj0911* mutants showed significantly reduced Cco activity consistent with these gene products being involved in assembly of the oxidase. There was a greater reduction of Cco activity in Δ*cj0908–cj0911* (∼40% activity of WT) than in Δ*cj0911* (∼60% activity of WT), suggesting the additional importance of *cj0908*, *cj0909*, and *cj0910* gene products but single deletions in each of these genes will be required to dissect their individual contributions. Work in other bacteria has shown that the periplasmic copper chaperones are required under conditions of low copper availability only and excess Cu in the medium often rescues the absence of either PCuC or Sco1 or both (Trasnea et al., 2016). When grown with excess Cu in the medium, Δ*cj0911* did show higher Cco activity but there was no difference in the Δ*cj0908–cj0911* strain. This suggests that it is not just the absence of both PCuC and Sco1 homologues in Δ*cj0908–cj0911* but also the absence of *cj0908* and/or *cj0910*, which is affecting the Cco activity. The paralogous Cj0908 and Cj910 proteins are predicted to be membrane anchored with a periplasmic domain containing Trx/Fd-like Cys motifs suggestive of a redox function. A possible role for these proteins could be to reduce Cu(II) to Cu(I), which is the form required for insertion into CcoN, prior to handling by the chaperones.

Cj1483 is a CcoH homologue based on BLAST searches. Δ*ccoH* has a growth defect and no Cco activity at all, irrespective of the amount of Cu in the medium. This observation is in accordance with the model suggested by Pawlik et al. (2010) in which biogenesis of *cbb*_3_-type Cco proceeds via CcoQP and CcoNO subcomplexes in *R. capsulatus*, but neither the fully assembled *cbb*_3_-type Cco nor the CcoQP or CcoNO subcomplex was detectable in cells lacking CcoH. They also showed that CcoH remains tightly associated with the active, fully assembled CcoNOQP complex.

We identified three novel genes located between *ccoH* (*cj1483c*) and *ccoP* (*cj1487c*), each of which was required for maximum oxidase activity. Mutant studies showed that CcoX (Cj1486) was essential for oxidase activity and maximum growth yield, while CcoY (Cj1485) and CcoZ (Cj1484) were important although not essential for oxidase activity. Fascinatingly, CcoZ has structural similarity (Supplementary Figure 1) to proteins that have been found to be associated with the cytochrome *bcc*-oxidase supercomplex in actinobacteria, e.g., *M. smegmatis* (Gong et al., 2018; Wiseman et al., 2018). Msmeg_4692 appears to mediate interactions of the oxidase with an extended C-terminal loop of the cytochrome *bcc* QcrB subunit (Wiseman et al., 2018). Examination of the QcrB sequence in *C. jejuni* does not show evidence of an unusually long C-terminal extension. However, one other unique aspect of the Qcr complex in the Campylobacterota in general and *C. jejuni* in particular, is that it contains a dihaem cytochrome *c* analogous to the situation in actinobacteria (Garg et al., 2018). Taken together, these observations raised the possibility that a Qcr-oxidase supercomplex might be present in *C. jejuni*, even though this bacterium contains multiple soluble periplasmic *c*-type cytochromes, two of which have been previously shown to be electron donors to the *cbb*_3_-oxidase (Liu and Kelly, 2015). However, there is now evidence from *Rhodobacter* that Gram-negative *cbb*_3_-type oxidases can form supercomplexes (Steimle et al., 2021). The presence of CcoZ in the same high molecular weight band resolved by BN-PAGE as the subunits of both the CcoNOP and QcrABC proteins is consistent with such an association in *C. jejuni*, but clearly further work will be needed to confirm this. Such a complex may be unstable and difficult to isolate, as obtaining the cryo-EM structure of the *Rhodobacter* supercomplex required artificial linking of the constituent Qcr and oxidase complexes (Steimle et al., 2021). Unlike the obligate Qcr-Cco interaction in actinobacteria (where soluble c-type cytochromes are absent), in Gram-negative bacteria there may be regulation of the association of these complexes (e.g., under different growth conditions or oxygen supply) and it would be informative to examine how electron transfer from Qcr to the Cco complex in *C. jejuni* is altered in the absence of CcoXYZ as well as how Qcr-oxidase interactions are changed.

Finally, a link between oxidase activity and copper homeostasis in *C. jejuni* was suggested by the reduced oxidase activity in mutants in the *copA* (*cj1161c*) and *copZ* (*cj1162c*) genes. Metal homeostasis in the Campylobacterota has recently been reviewed (Kelley et al., 2021) but copper homeostasis is still incompletely understood. The copper sensitive phenotype of a *copA* mutant has been established previously (Hall et al., 2008) and such a mutant is also oxidative stress sensitive (Gardner and Olson, 2018). The function of the *cj1162c* gene has not been previously characterized but our results show that this *copZ* homologue also protects against copper toxicity; its expression is increased by exogenous copper and its deletion phenocopies that of a *copA* mutant; increased intracellular copper and copper sensitive growth. One explanation for the reduced oxidase activity in these mutants is that an inability to regulate intracellular Cu disturbs the correct copper trafficking though CcoI, necessary for oxidase assembly. Interestingly, in *R. capsulatus*, where deletion of *copZ* also results in lower *cbb*_3_-oxidase activity, a membrane bound complex between CopZ and CcoI was detected, suggesting that the CopZ chaperone can supply CcoI with copper, as well as interacting with CopA for copper detoxification (Utz et al., 2019). However, in *C. jejuni*, the deletion of *ccoI* led to abolition of oxidase activity whereas deletion of *copZ* resulted in only a ∼30% reduction compared to wild-type in the absence of excess copper and ∼50% reduction in the presence of copper, so the phenotypes are not identical, as might be expected for an obligate CcoI-CopZ complex.

**FIGURE 8:**
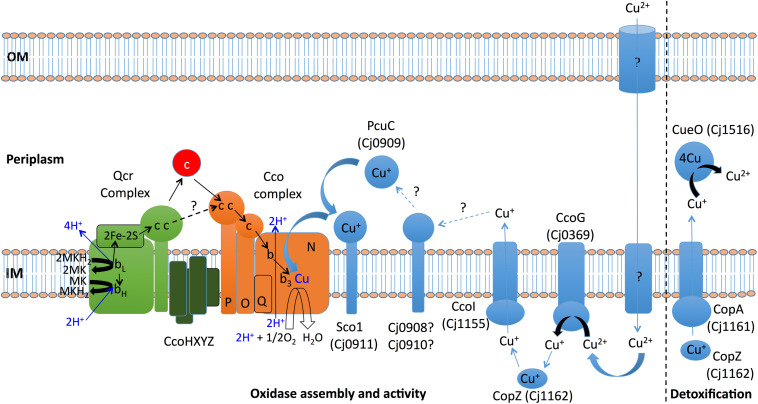
Model for copper handling and homeostasis in *C. jejuni* NCTC 11168. Copper must be transported from the environment into the cell, but the mechanism is currently unknown as there is no CcoA importer in *C. jejuni*. Copper homeostasis/detoxification is achieved by the combined action of the CopA ATP-driven efflux pump, receiving copper from the cytoplasmic chaperone CopZ and the periplasmic multi-copper oxidase CueO (Cj1516) which oxidises toxic cupric ions to less toxic cupric ions. CcoG (Cj0369) acts as a reductase that probably passes cuprous ions to CopZ. CpoZ is also thought to interact with CcoI, a specific efflux pump that is essential for copper delivery to the periplasm for transfer to the Cco enzyme complex, possibly via potential reductases Cj0908 and Cj0910 and copper chaperones PcuC (Cj0909) and Sco1 (Cj0911). Sco1 delivers cuprous copper to the CcoN subunit of the oxidase complex to form the bi-nuclear catalytic centre of the enzyme. Newly identified genes in this study encode three proteins (CcoXYZ) that might interact with both the Qcr and oxidase, perhaps forming a supercomplex. Direct copper handling proteins are shown in light blue, with light blue arrows depicting the transfer of copper ions. Electron transfers are shown as black arrows. Dark blue arrows show proton binding and release or direct proton translocation associated with electron transfer from Qcr to the oxidase complex. Soluble cytochromes *c* in the periplasm are shown as a red circle. The possibility of direct electron transfer between Qcr and oxidase is indicated by a dashed black line. OM, outer membrane; IM, inner (cytoplasmic) membrane.

Based on the results obtained in this study, Figure 8 shows a working model for the handling of copper in *C. jejuni* NCTC 11168, particularly in relation to the assembly and function of the Cco enzyme. The major unanswered questions concern the nature of the pathway for import of copper, the role of the Cj0908/Cj0910 and CcoXYZ proteins, and the possibility of Qcr-oxidase supercomplex formation.

## Data Availability Statement

The original contributions presented in the study are included in the article/Supplementary Material, further inquiries can be directed to the corresponding author.

## Author Contributions

DK designed the study and wrote the manuscript. NG, AT, FP, SF, and PJ performed the experiments and analysed the data. DK, PJ, and MJ supervised the work and edited the manuscript. All authors contributed to the article and approved the manuscript.

## Conflict of Interest

The authors declare that the research was conducted in the absence of any commercial or financial relationships that could be construed as a potential conflict of interest.

## Footnotes

1 https://www.uniprot.org/proteomes/UP000249026
