# Supplementary material for: Genes Linking Copper Trafficking and Homeostasis to the Biogenesis and Activity of the cbb3-Type Cytochrome c Oxidase in the Enteric Pathogen Campylobacter jejuni
Source: Front Microbiol. 2021 Jun 25;12:683260. doi: 10.3389/fmicb.2021.683260 (PMC8267372; doi:10.3389/fmicb.2021.683260)
Supplement: Supplementary file 1 [file Data_Sheet_1.pdf]

SUPPLEMENTARY FIGURES 1-3

Genes linking copper trafficking and homeostasis to the biogenesis and activity of the *cbb*$_3$-type cytochrome *c* oxidase in the enteric pathogen *Campylobacter jejuni*

Nitanshu Garg, Aidan J. Taylor, Federica Pastorelli, Sarah E. Flannery, Phillip J. Jackson, Matthew P. Johnson and David J. Kelly*

Department of Molecular Biology and Biotechnology, The University of Sheffield, Firth Court, Western Bank, Sheffield S10 2TN, UK.

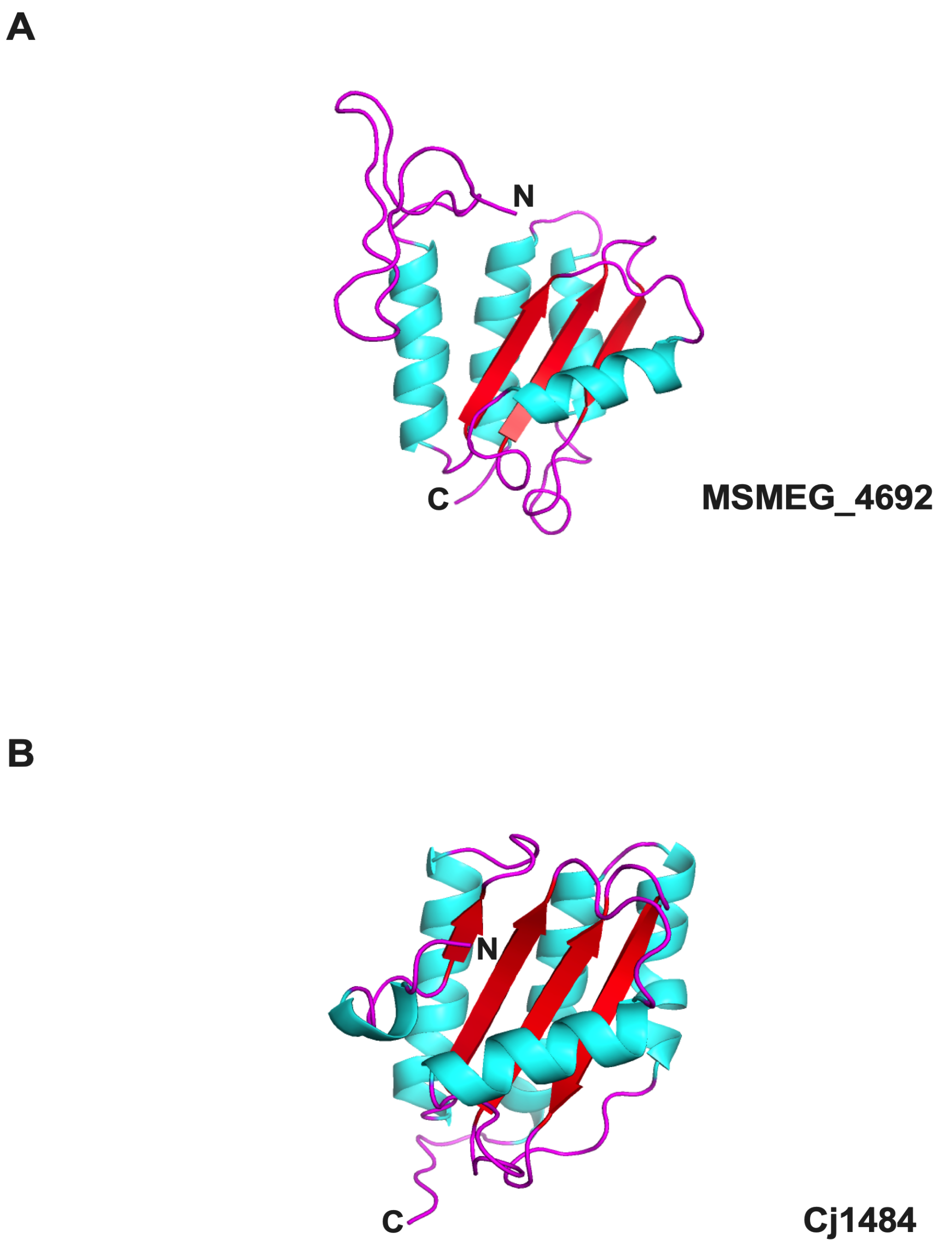

Figure S1. CcoZ (Cj1484) shows structural similarity to MSMEG-4692. **(A)** shows the actual 3D-stucture of MSMEG_4692 (PDB entry 6HWH entity 9) determined by Wiseman *et al*. (2018). (**B**) shows the result of modelling the Cj1484 sequence on MSMEG_4692 using the Phyre2 server (Kelley *et al*., 2015).

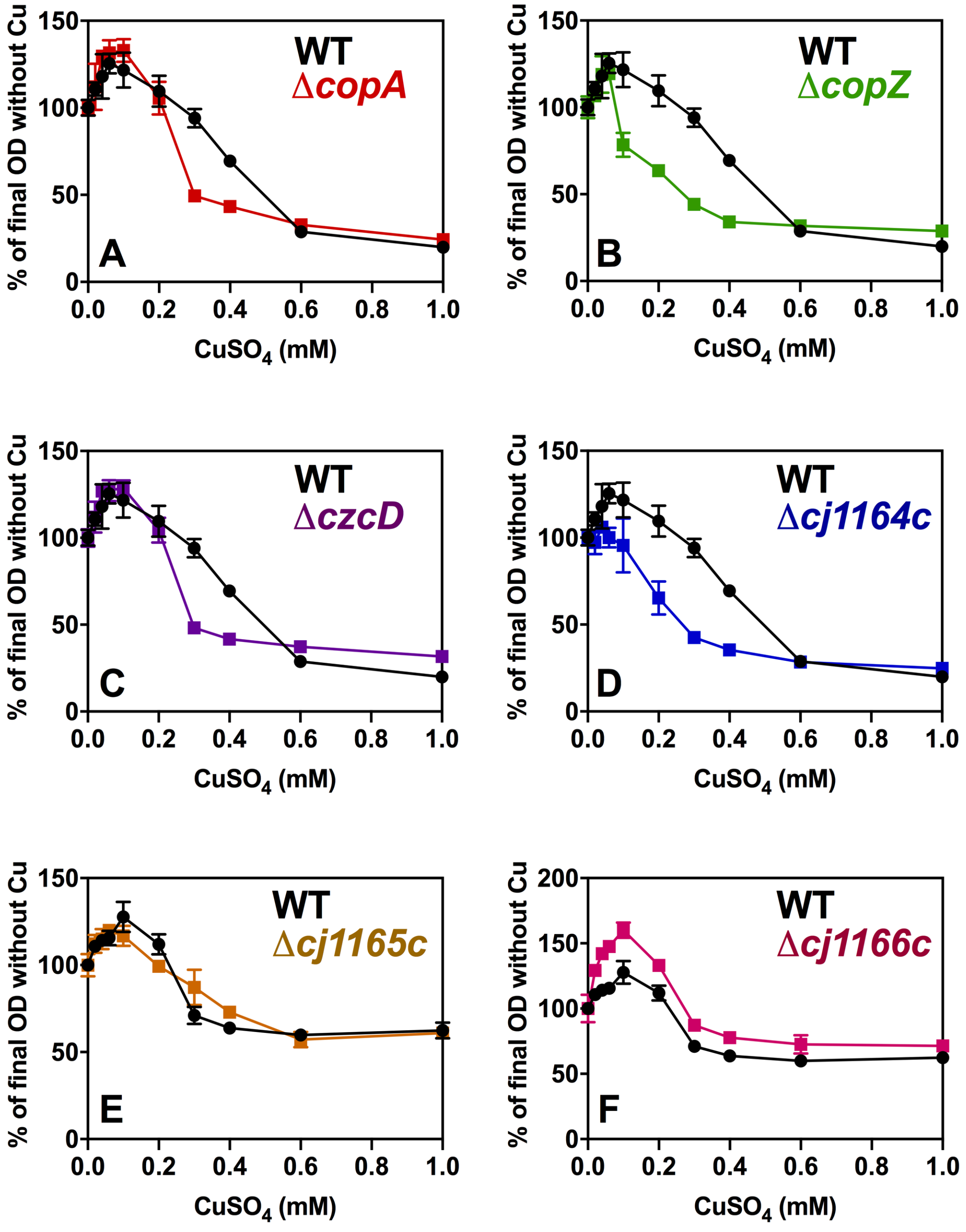

Figure S2. **Copper sensitivity profiles of individual deletion mutants in the *cj1161c-cj1166c* gene cluster**. Cells were grown in minimal media for 24h at the range of copper sulphate concentrations shown and the final OD 600 nm values compared with those of cells grown without added copper. The data points are derived from the mean of three independent cultures expressed as a percentage of the mean final OD of the zero added copper control cultures. The raw OD data are shown in Supplementary Table 1. Note that the WT control cultures are the same for panels A-D, while separate cultures were set up for Panels E-F as these data were obtained on a different day.

A

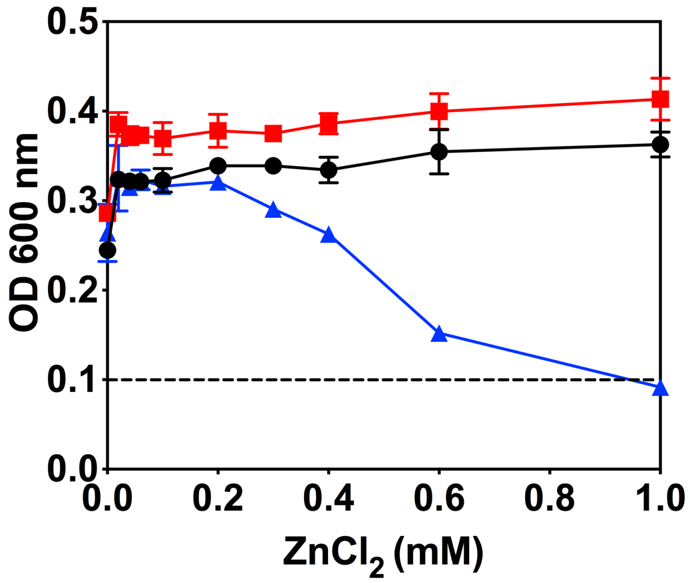

B

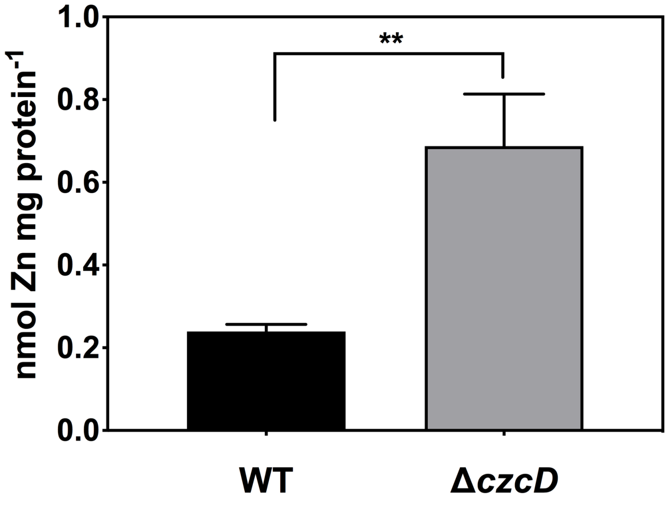

Figure S3. A *czcD* (*cj1163c)* mutant is sensitive to zinc and has an increased intracellular zinc content. In **(A)** cells of wild-type (black circles), *copA* mutant (red squares) or *czcD* mutant (blue triangles) were grown in minimal media for 24h with the range of zinc concentrations shown and the final OD 600 nm values determined. The data points are the mean with error bars showing the SD of three independent cultures for each metal salt concentration. The dotted line represents the starting OD of the cultures. Panel **(B)** shows the intracellular zinc content measured by ICP-MS, normalised to total cell protein of wild-type (WT) or *czcD* mutant. The data are the means and SD of three independent cultures, grown in minimal media without added zinc. ** p = 0.0036 by Student's t-test.
